# Embedded CRISPRi Enhances Gene‐Silencing Efficiency in *Drosophila*

**DOI:** 10.1002/advs.202515849

**Published:** 2026-03-20

**Authors:** Pengchong Fu, Xuedi Zhang, Ying Zhou, Jie Zheng, Angyang Sun, Kailong Zhuang, Weiwei Bao, Guanjun Gao

**Affiliations:** ^1^ School of Basic Medical Sciences Suzhou Medical College Soochow University Suzhou Jiangsu Province China; ^2^ Gene Editing Center School of Life Science and Technology ShanghaiTech University Shanghai China

**Keywords:** CRISPRi, dCas9, drosophila, transcriptional repression

## Abstract

CRISPR interference (CRISPRi), leveraging catalytically inactive Cas9 (dCas9), has transformed transcriptional silencing. However, its application in *Drosophila melanogaster* has been constrained by inconsistent efficiency and limited repression amplitude. Here, we present embedded CRISPR interference (emCRISPRi), an advanced gene‐silencing platform that integrates transcriptional repression domains (Mxi and TRD) into a structurally flexible region of dCas9. This design significantly enhances silencing efficiency, enabling robust repression of coding genes and cis‐regulatory elements, particularly at transcription start site (TSS)‐proximal regions. emCRISPRi demonstrates improved gene‐silencing activity compared to RNA interference (RNAi) at several tested loci and facilitates strong phenotypic rescue via unmodified cDNA. Its versatility is demonstrated through the dissection of Hippo pathway interactions and the mitigation of TDP‐43‐induced neurotoxicity in an amyotrophic lateral sclerosis (ALS) model. These findings position emCRISPRi as a transformative tool for functional genomics, enhancer studies, and disease modeling in *Drosophila*, with significant potential for cross‐species adaptation and therapeutic innovation.

## Graphical abstract

Current CRISPR interference (CRISPRi) technology in Drosophila has limited efficiency. This study introduces the emCRISPRi platform, which significantly enhances transcriptional silencing efficacy by embedding inhibitory domains within the dCas9 architecture. Moreover, emCRISPRi can be applied to target enhancers and disease models, offering a robust and versatile tool for precision functional genomics.

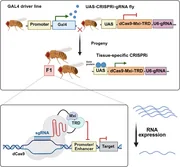

## 1 Introduction

The CRISPR/Cas system, derived from the adaptive immune mechanisms of prokaryotes, has transformed genetic engineering by enabling programmable targeting of nucleic acids [1]. Its versatility allows for a wide range of applications, including genome editing, base editing, and transcriptional regulation across diverse organisms [2, 3, 4]. Among the CRISPR/Cas protein variants, Cas9 is the most widely used due to its RNA‐guided endonuclease activity, which is mediated by its RuvC and HNH nuclease domains [5]. By inactivating these nuclease domains, a catalytically inactive form (dCas9) is generated, which retains DNA‐binding ability without cleaving DNA. This modified protein has emerged as powerful scaffold for recruiting transcriptional regulators or epigenetic modifiers, forming the basis of CRISPR interference (CRISPRi) for gene silencing and CRISPR activation (CRISPRa) for gene upregulation [6, 7].

CRISPRi employs dCas9 fused to transcriptional repression domains, such as the KRAB family domains (e.g., ZNF10, Zim3) or epigenetic suppressors like MeCP2, to inhibit transcriptional machinery or modify chromatin, leading to targeted gene silencing [8]. Compared to RNA interference (RNAi), which degrades mRNA at the post‐transcriptional level [9], CRISPRi operates at the DNA level, facilitating functional rescue using unmodified cDNA [5, 10]. These characteristics have made CRISPRi an invaluable tool for dissecting gene function, studying transcriptional regulation, and mapping regulatory networks in a variety of model systems, including bacterial, mammalian cells, and other organisms. However, in *Drosophila melanogaster*, a premier organism for developmental biology and human disease studies, the application of CRISPRi has been hindered by challenges such as suboptimal silencing efficiency [11, 12].

As a cornerstone model for functional genomics, *Drosophila* offers unique advantages, including a short generation time, extensive genetic toolkit, and a high degree of genomic conservation with humans [13]. Traditionally, RNAi has been the dominant method for gene silencing in *Drosophila*, but its use is often hindered by incomplete silencing and off‐target effects, which can limit reproducibility in functional studies [14]. Recently, Cas13‐based RNA‐targeting approaches have emerged as an alternative to RNAi. Among these, RfxCas13d has demonstrated strong cleavage activity and notable knockdown efficiency in *Drosophila* [15]. However, a major limitation of Cas13 systems, including RfxCas13d, is their inherent trans‐cleavage activity, an intrinsic biochemical property of the Cas13 family, leads to elevated off‐target effects in *Drosophila* cells and restricts the broader applicability of Cas13‐mediated knockdown [16]. CRISPRi offers an attractive alternative due to its ability to silence genes without generating double‐strand breaks [17]. While efforts to optimize CRISPRi in *Drosophila* have shown progress, challenges remain. Codon optimization of dCas9 has improved its expression and silencing efficiency in *Drosophila* tissues [11], and conditional dCas9 systems have been developed to enable tissue‐specific repression, which is particularly useful for spatially restricted gene functions [18]. dCas9‐KRAB fusions have been applied to repress retrotransposons, revealing their utility in aging and lifespan studies [19]. Truncated guide RNAs (tgRNAs) have been developed to enable precise targeting of enhancers and promoters without inducing DNA breaks [12]. Additionally, fusion of dCas9 with repressive proteins like CtBP and Rbf has provided moderate improvements in silencing efficiency [20, 21]. However, the full potential of CRISPRi in *Drosophila* has not yet been realized, and further advancements are needed to overcome existing limitations.

Protein engineering provides a robust framework for both directed evolution and the rational modification of protein function [22, 23]. Circular permutation, a form of topological rearrangement, has been employed to modulate SpCas9's properties, including its susceptibility to protease activity [24]. For instance, the 198–206 loop has been identified as a permissive site for structural modifications. Similarly, split‐Cas9 systems have been developed by cleaving flexible regions within the protein, enabling controllable and modular applications [25, 26]. Furthermore, insertion or substitution of base‐editing domains into these flexible loops has led to the development of more precise and versatile base‐editing tools [27, 28, 29, 30, 31]. Despite these advancements, the potential of a CRISPRi platform utilizing an embedded architecture within the SpCas9 protein itself has not yet been explored in *Drosophila*.

Transcriptional repression domains, such as KRAB, TRD of MeCP2, and Mxi, have shown promise in enhancing gene silencing in mammalian systems [8, 32]. Inspired by these findings, we hypothesized that either directly fusing these repression domains to dCas9 or embedding them within the dCas9 protein structure could provide a more efficient and effective CRISPRi platform for *Drosophila*.

In this study, we introduce domain‐embedded CRISPR interference (emCRISPRi), an innovative gene‐silencing platform designed to address the limitations of conventional CRISPRi systems. To enhance repressive activity, we incorporate the Mxi and MeCP2‐TRD domains, both of which recruit histone deacetylase complexes [33, 34, 35], leading to histone deacetylation, chromatin compaction, and robust transcriptional silencing [36]. By embedding Mxi and TRD domains into a structurally flexible region of dCas9, emCRISPRi achieves significantly improved transcriptional suppression efficiency compared to dCas9 alone or conventional fusion designs. This system enables precise targeting of cis‐regulatory elements, such as enhancers, without disrupting surrounding sequences, and supports simultaneous silencing of multiple genes. We demonstrate the utility of emCRISPRi in dissecting complex genetic interactions, studying enhancer function, and modeling human diseases, establishing it as a transformative tool for functional genomics and regulatory biology in *Drosophila*.

## 2 Results

### 2.1 Structural Embedding of Repressor Domains in dCas9 Enhances Silencing Efficiency in *Drosophila*

To investigate whether embedding transcriptional repression domains within dCas9 improves gene‐silencing efficiency, we utilized *Drosophila melanogaster* as a model organism and targeted the well‐characterized *w* gene, which encodes a marker for eye pigmentation [37]. Wild‐type flies exhibit red compound eyes, whereas silencing or disrupting *w* results in lighter or white eyes, providing a visually interpretable readout for transcriptional repression [38].

**FIGURE 1:**
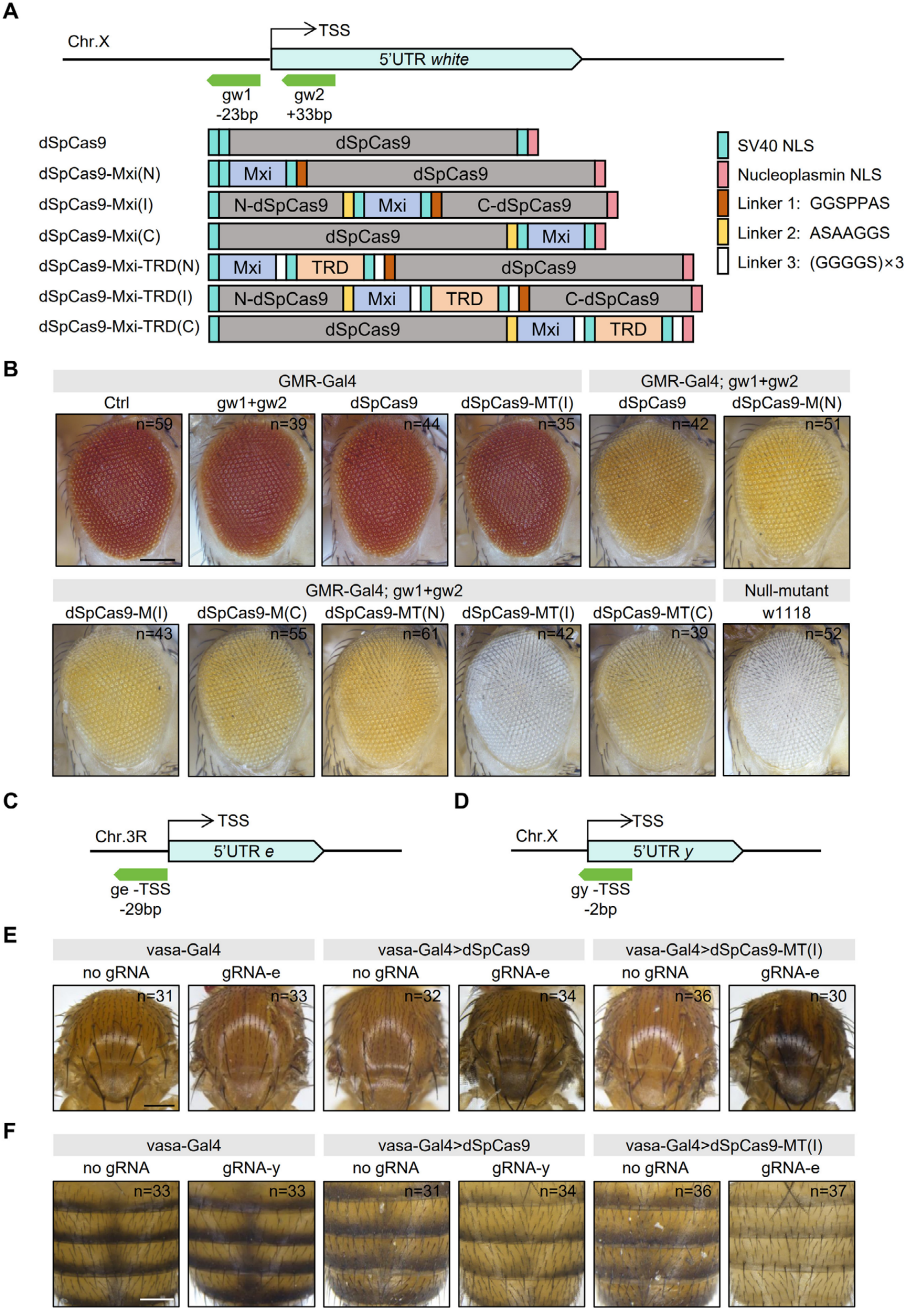
The embedded repressor domain within dSpCas9 enhances gene‐silencing efficiency. (A) Schematic illustration of the CRISPRi system designed for transcriptional repression in *Drosophila melanogaster*. The genomic locus of the *white* gene on the X chromosome is shown, highlighting the positions of two designed sgRNAs (gw1 and gw2) designed near the transcription start site (TSS). The CRISPRi protein components are color‐coded to denote specific elements: Cornflower Blue (SV40 nuclear localization signal, NLS), Scarlet (Nucleoplasmin NLS), Brown (Linker 1), Yellow (Linker 2), White (Linker 3), Gray (dSpCas9, codon‐optimize for *Drosophila melanogaster*), Light Blue (Mxi repression domain), and Chocolate Yellow (TRD of MeCP2, transcriptional repression domain). (B) Microscopic images of *Drosophila* adult eyes showing the inhibitory effects of CRISPRi protein variants. The proteins were expressed under the control of the GMR‐Gal4 driver, and the gRNAs (gw1 and gw2) were driven by the U6:1 and U6:3 promoters, respectively. Images are representative of biological replicates, with the specific sample size (n) for each group indicated in the corresponding panels. Scale bar: 100 µm. (C) The genomic locus of the *e* gene on chromosome 3R is depicted, indicating the position of the TSS‐proximal gRNA (ge‐TSS). (D) The genomic locus of the *y* gene on chromosome X is shown, with the TSS‐proximal gRNA (gy‐TSS) position indicated. (E) Microscopic images of *Drosophila* adult dorsal thoracic structures showing the phenotypic comparison of *e* gene silencing by dSpCas9 and dSpCas9‐MT(I). The proteins were expressed under the control of the vasa‐Gal4 driver. Images are representative of biological replicates, with the specific sample size (n) for each group indicated in the panels. Scale bar: 200 µm. (F) Microscopic images of *Drosophila* adult abdomen (dorsal view) showing the phenotypic comparison of *y* gene silencing by dSpCas9 and dSpCas9‐MT(I). The proteins were expressed under the control of the vasa‐Gal4 driver. Images are representative of biological replicates, with the specific sample size (n) for each group indicated in the panels. Scale bar: 200 µm.

Drawing on evidence from mammalian studies where repressor domains (e.g., KRAB, Mxi and the transcriptional repression domain (TRD) of MeCP2) improved silencing efficiency [8, 32], we hypothesized that embedding these domains within dCas9 or fusing them to dCas9 would similarly enhance transcriptional repression in *Drosophila*. To test this, we designed two gRNAs targeting sequences near the transcription start site (TSS) of w (−23 to +33 bp) (Figure 1A), as proximity to the TSS has been shown to enhance silencing efficiency [39]. All gRNAs were ubiquitously expressed under the U6 promoter.

Based on codon‐optimized sequences of both the dCas9 scaffold and the repressor domains tailored to *Drosophila melanogaster* [11], we engineered dCas9 variants fused with transcriptional repression domains in five configurations: N‐terminal, C‐terminal, and three embedded configuration where repressor domains were embedded within structurally flexible region of dCas9 (residues V206‐D207, L229‐P230, S1248‐P1249, identified through structural modeling) [5, 24] (Figure 1A; Figure S1). These constructs were expressed in the eye using the *GMR*‐*Gal4* driver to evaluate gene‐silencing efficiency [40] (Figure S2), with reductions in eye pigmentation serving as a functional readout (Figure 1B).

Native dCas9 (without repression domains) produced a modest reduction in eye pigmentation, likely due to steric hindrance at the TSS caused by dCas9 binding, which obstructs RNA polymerase activity (Figure 1B). In contrast, fusing the Mxi repressor domain to dCas9 significantly improved silencing. Among the tested configurations, the embedded Mxi construct at the V206‐D207 site exhibited the highest repression efficiency, outperforming both N‐ and C‐terminal fusions. We designated this fusion variant dCas9‐M(I) (Figure 1B). Building on this improvement, we incorporated the TRD of MeCP2 into the embedded Mxi (V206‐D207) construct, generating a dual‐repressor fusion, dCas9‐Mxi‐TRD, hereafter referred to as dCas9‐MT. Flies expressing dCas9‐MT with the V206‐D207 embedded configuration exhibited near‐complete loss of eye pigmentation, producing phenotypes comparable to genetic null mutants. We named this optimized variant dCas9‐MT(I) (Figure 1B).

Furthermore, quantitative fluorescence PCR (qPCR) demonstrated that dCas9‐MT(I) achieved higher levels of transcriptional repression compared to other fusion constructs (Figures S3 and S4). In contrast, the alternative intronic embedding sites (L229‐P230 and S1248‐P1249) failed to produce satisfactory repression, underscoring the specificity and effectiveness of the V206–D207 site as the optimal location for embedding repressor domains (Figure S3).

After identifying V206–D207 as the optimal embedding site, we next evaluated the performance of the KRAB repressor domain. However, KRAB‐dCas9 constructs, regardless of the configurations (N‐terminal, C‐terminal, or V206–D207 embedded), failed to significantly enhance silencing efficiency (Figure S5). These results suggest that KRAB domain is ineffective for transcriptional repression in *Drosophila*.

To further validate the efficacy of dCas9‐MT(I), we selected *hippo* (*hpo*) as an additional target gene. Two gRNAs targeting regions proximal to the *hpo* transcription start site (−33 bp to −13 bp) were designed and expressed under the control of the U6 promoter (Figure S6A). The same set of dCas9 fusion variants was systematically evaluated using the *ey‐Gal4* driver [41]. Consistent with the results obtained for *white*, the embedded configuration (dCas9‐MT(I)) consistently outperformed both N‐ and C‐terminal fusions when targeting *hpo*. These findings underscore the structural and functional advantages of embedding repressor domains directly within dCas9, particularly at the V206–D207 site (Figure S6B,C).

Collectively, these findings establish the embedded CRISPRi (emCRISPRi) system as a superior platform for targeted gene silencing in *Drosophila*. While Mxi and TRD domains contribute individually to repression, their combination within the embedded construct (dCas9‐MT(I)) produced a synergistic effect, achieving robust transcriptional silencing.

### 2.2 Extending emCRISPRi to Additional Targets

To evaluate the versatility of the embedded CRISPRi (emCRISPRi) system, we targeted two pigmentation‐related genes in *Drosophila*: *ebony* (*e*) and *yellow* (*y*). The *e* gene suppresses melanin formation, and its disruption leads to systemic dark pigmentation [42]. In contrast, the y gene promotes black pigment accumulation, and its disruption results in a yellow cuticle phenotype [43, 44].

We designed gRNAs targeting sequences near the TSS of *e* (−29 bp) and *y* (−2 bp) (Figure 1C,D). Native dCas9 or dCas9‐MT(I) was expressed using the *vasa*‐*Gal4*, which drives low‐level ubiquitous expression in somatic cells and higher expression in the germline [45]. Pigmentation phenotypes were then analyzed to assess the functional consequences of gene repression in these tissues. Both constructs induced the expected pigmentation loss‐of‐function phenotypes for e and y, but flies expressing dCas9‐MT(I) exhibited significantly stronger phenotypic effects compared to those expressing native dCas9 (Figure 1E,F). Specifically, dCas9‐MT(I) nearly abolished black pigmentation in *y*‐targeted flies and caused a more pronounced dark pigmentation phenotype in *e*‐targeted flies. To further validated these observations, we performed qRT‐PCR, which confirmed that dCas9‐MT(I) achieved significantly stronger transcriptional repression compared to native dCas9 (Figure S7A,B). These results highlight the broad applicability of emCRISPRi for silencing diverse target genes with enhanced efficiency.

### 2.3 Achieving Knockout‐Level Silencing While Maintaining Normal Viability

To determine whether emCRISPRi could achieves silencing efficiency comparable to genetic knockouts, we ubiquitously expressed the dCas9‐MT(I) construct using the *Act5C*‐*Gal4* driver and compared its effect on w to that of a complete *w* null mutant (w1118). Expression of dCas9‐MT(I) produced a phenotype indistinguishable from the w1118 null mutant, resulting in a near‐complete loss of eye pigmentation (Figure S8A).

To evaluate systemic safety, we monitored the viability of flies ubiquitously expressing dCas9‐MT(I) under the same conditions. The survival rates of these flies were comparable to those of wild‐type controls, indicating that high‐level and widespread expressed of emCRISPRi did not negatively impact the eclosion survival rate of F1 progeny (Figure S8B,C).

### 2.4 Silencing Multiple Genes Across Diverse Biological Pathways

To assess the robustness and versatility of emCRISPRi, we tested its ability to silence eight developmentally critical genes (dCUL1 [46], G6PD [46]*, Hel25E* [47], *Hsp110* [48], *Lon* [49], *MEP‐1* [50], *Rpt2* [51], and *SUV39* [52]), which are involved in processes such as cell cycle regulation, metabolic balance, protein homeostasis, and chromatin modifications.

**FIGURE 2:**
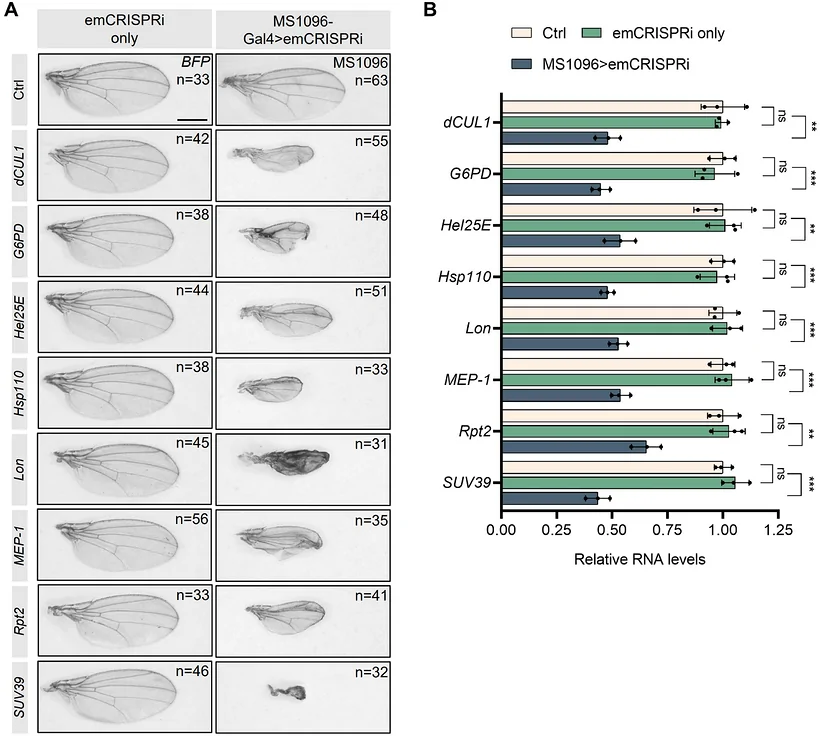
emCRISPRi achieves robust and efficient gene silencing across multiple targets (A) Microscopic images of *Drosophila* adult wings showing the phenotypic analysis of gene silencing by emCRISPRi in vivo. A range of endogenous genes was targeted with two gRNAs, expressed under the control of the MS1096‐Gal4 driver. Images are representative of biological replicates, with the specific sample size (n) for each group indicated in the panels. Scale bar: 500 µm. (Ctrl: BFP: gRNA targeting blue fluorescent protein; MS1096: MS1096‐Gal4/ +). (B) Quantitative RT‐qPCR analysis of transcript levels for target genes silenced by emCRISPRi under *MS1096‐Gal4* control. The housekeeping gene *rp49* was used as an internal reference for normalization. Each data point in the bar graph represents an independent biological replicate, derived from the average of two technical replicates. Data are presented as mean ± SD (n = 3 biological replicates). Statistical analysis was performed using one‐way ANOVA with Tukey's honestly significant difference (HSD) test in GraphPad Prism. ** indicates *p* < 0.01. *** indicates *p* < 0.001.

Tissue‐specific gene silencing was achieved by expressing dCas9‐MT(I) using the wing disc‐specific *MS1096*‐*Gal4* driver [53, 54]. Phenotypic analysis revealed detectable developmental defects for all eight genes, with severity varying between targets. Mild abnormalities were observed for genes such as *Hel25E* and *Rpt2*, while more pronounced morphological disruptions were noted for *G6PD* and *SUV39* (Figure 2A; Figure S9). qRT‐PCR analysis confirmed transcriptional knockdown levels of 30%–60% for most genes, with four genes showing knockdown efficiencies exceeding 50%. Notably, *SUV39* exhibited the highest knockdown efficiency (Figure 2B). These results demonstrate that emCRISPRi can effectively silence a diverse array of genes, underscoring its utility as a tool of functional genomics studies in *Drosophila*.

### 2.5 Comparison Gene‐Silencing Efficiency: EmCRISPRi and RNAi

To benchmark the gene‐silencing efficiency of emCRISPRi against RNAi, we selected 10 target genes for parallel analysis. RNAi constructs targeted mRNA regions, while emCRISPRi gRNAs were designed to target sequences near the TSS. Both methods utilized the wing‐specific MS1096‐Gal4 driver for tissue‐specific expression, with U6 promoters driving gRNA expression in emCRISPRi.

**FIGURE 3:**
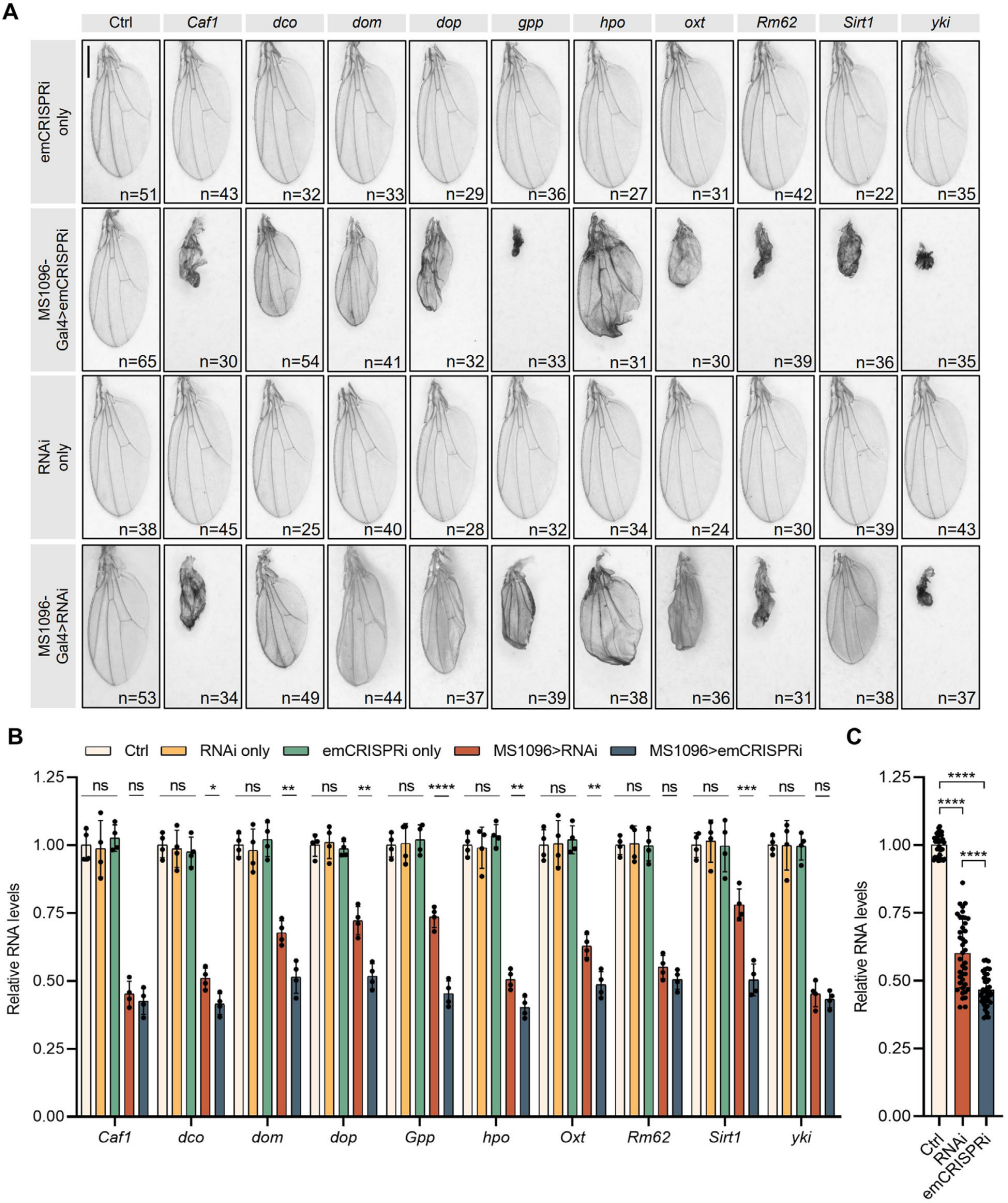
emCRISPRi outperforms RNAi in gene‐silencing efficiency (A) Microscopic images of *Drosophila* adult wings showing the phenotypic comparison of gene silencing by emCRISPRi and RNAi across multiple gene loci. All constructs were driven by the MS1096‐Gal4 driver. Images are representative of biological replicates, with the specific sample size (n) for each group indicated in the panels. Scale bar: 500 µm. (B) Quantitative RT‐qPCR analysis comparing the gene‐silencing efficiency of emCRISPRi and RNAi at different gene loci. Transcript levels of target genes were measured, with rp49 serving as the internal reference for normalization. Each data point in the bar graph represents an independent biological replicate, derived from the average of two technical replicates. Data are presented as mean ± SD (n = 4 biological replicates). Statistical analysis was performed using one‐way ANOVA with Tukey's honestly significant difference (HSD) test in GraphPad Prism. *, **, *** and **** indicate *p* < 0.05, *p* < 0.01, *p* < 0.001 and *p* < 0.0001, respectively, and ns indicates no significance. (C) Summary of gene‐silencing efficiency based on the data in panel (b). emCRISPRi demonstrated significantly higher silencing efficiency compared to RNAi across the majority of tested loci, as indicated by reduced transcript levels. Data are presented as mean ± SD (n = 40). Statistical analysis was performed using one‐way ANOVA with Tukey's honestly significant difference (HSD) test in GraphPad Prism. *, **, *** and **** indicate *p* < 0.05, *p* < 0.01, *p* < 0.001 and *p* < 0.0001, respectively, and ns indicates no significance.

Phenotypic analysis of the wing morphology revealed that emCRISPRi consistently produced robust and reproducible developmental defects across all tested genes, comparable to the phenotypes observed with RNAi (Figure 3A; Figure S10). Notably, for several genes such as dop, gpp, and sirt1, emCRISPRi caused significantly more severe wing phenotypic defects than RNAi, which induced only moderate changes for these genes.

To further quantify gene‐silencing efficiency, qRT‐PCR was performed on wing tissues for target genes. Statistical analysis revealed that for 7 of 10 genes, emCRISPRi exhibited significantly stronger silencing than RNAi (Figure 3B). Specially, for targets such as dop, gpp, and sirt1, emCRISPRi achieved higher knockdown efficiencies, which correlated with more pronounced phenotypic defects observed (Figure 3A, B and Figure S10). These findings establish emCRISPRi as not only a highly effective gene‐silencing platform but also one that demonstrates greater consistency and, in some cases, superior efficiency compared to RNAi. Together, the results underscore the potential of emCRISPRi as a powerful alternative to RNAi for functional genetic studies in Drosophila (Figure 3C).

### 2.6 cDNA‐Based Phenotypic Rescue of CRISPRi

To evaluate the specificity of emCRISPRi, we performed cDNA rescue experiments for three genes (yki, dco and hpo). Unlike RNAi, which requires the use of modified rescue constructs to prevent degradation of the rescue transcript, emCRISPRi targets genomic regions outside the coding sequence. This unique feature allows the use of unmodified cDNA, preserving the native structure and functionality of the transcript.

Co‐expression of emCRISPRi with unmodified cDNA, driven by MS1096‐Gal4, largely rescued the phenotypic defects caused by gene silencing across all three tested genes at the phenotypic level. (Figure S11A,B). These results show that CRISPRi enables phenotypic rescue using unmodified cDNA, thereby simplifying the validation of gene‐specific knockdowns.

### 2.7 Efficiency of emCRISPRi Across Diverse Core Promoter Types

Core promoters, spanning approximately −40 to +40 nucleotides relative to the TSS, plays a critical role in transcription initiation [55, 56] and are characterized by distinct DNA motifs, such as the TATA box [57, 58], initiator (Inr) [59, 60], downstream core promoter element (DPE) [61, 62], and others [44, 63, 64, 65, 66, 67]. These motifs contribute to the functional diversity of core promoters.

To investigate whether emCRISPRi can efficiently suppress transcription across diverse core promoter types, we classified the target genes analyzed in Figures 2 and 3 based on their promoter sequence features. Promoter classifications were informed by previous studies of Drosophila core promoter architecture (Figure S12A,B) [62, 68, 69].

Our analysis demonstrated that emCRISPRi achieved effective transcription repression across all tested promoter types, including those containing TATA box, DPE, and Inr motifs. No significant differences in suppression efficiency were observed between genes with different core promoter architectures, suggesting that the emCRISPRi system is broadly effective regardless of core promoter composition (Figure S12C). These findings further underscore the versatility of emCRISPRi as a gene‐silencing platform.

### 2.8 Precise Silencing Within Bidirectional Promoter Regions

**FIGURE 4:**
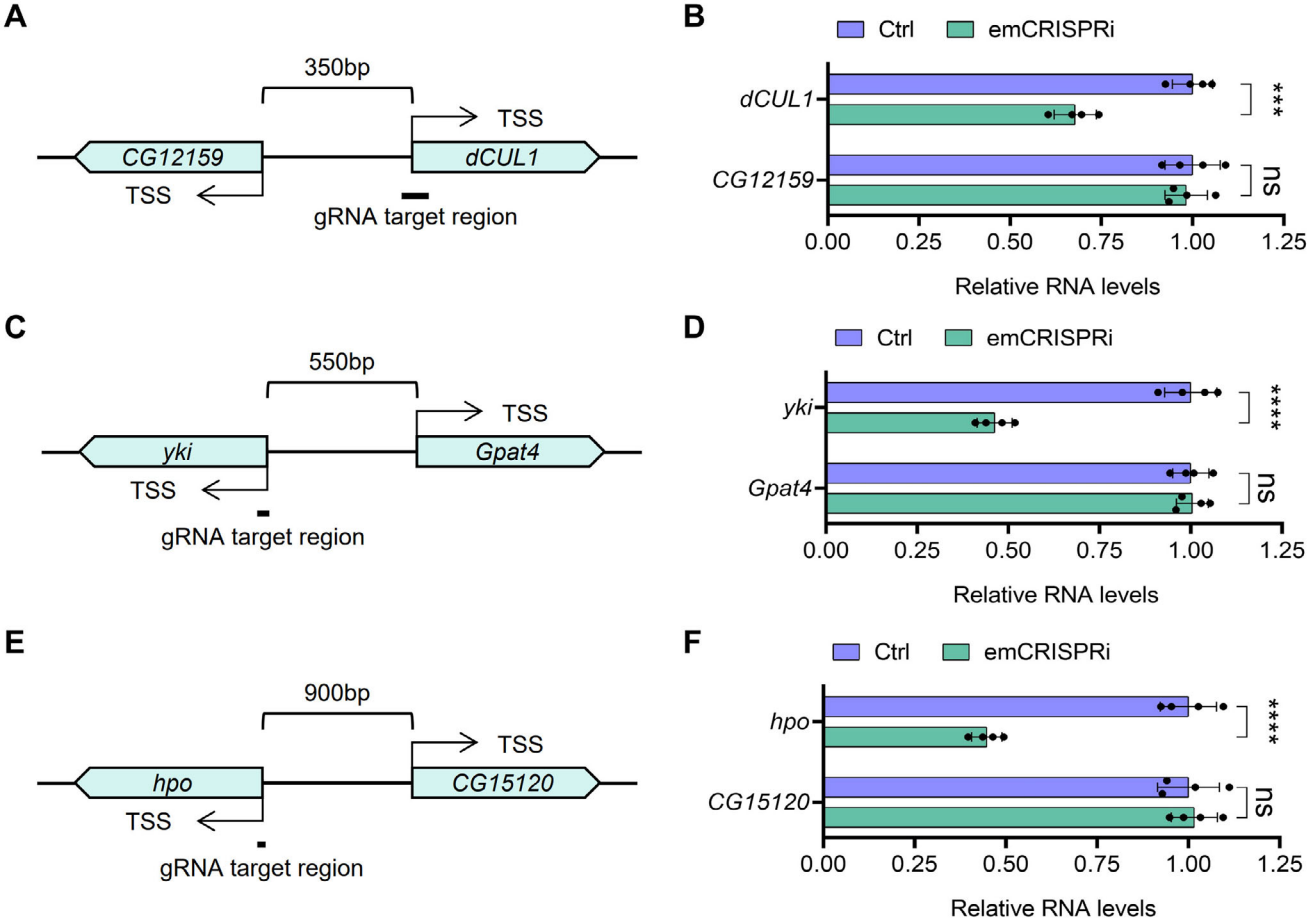
emCRISPRi achieves precise and specific targeting in bidirectional promoters’ region (A) Genome map illustrating the loci near the transcription initiation sites of *dCUL1* and *CG12159*. (B) Quantitative RT‐qPCR analysis of *dCUL1* and *CG12159* transcript levels following emCRISPRi targeting of *dCUL1*. Each data point in the bar graph represents an independent biological replicate, derived from the average of two technical replicates. Data are presented as mean ± SD (n = 4 biological replicates). Statistical significance was determined using two‐tailed Student's t‐test in GraphPad Prism. ***, *p* < 0.001; ns indicates no significance. (C) Genome map depicting the transcription initiation sites of *yki* and *Gpat4*. (D) Quantitative RT‐qPCR analysis of *yki* and *Gpat4* transcript levels following emCRISPRi targeting of *yki*. Each data point in the bar graph represents an independent biological replicate, derived from the average of two technical replicates. Data are presented as mean ± SD (n = 4 biological replicates). Statistical significance was determined using two‐tailed Student's *t‐*test in GraphPad Prism. ****, *p* < 0.0001; ns indicates no significance. (E) Genome map illustrating the loci near the transcription initiation sites of *hpo* and *CG15120*. (F) Quantitative RT‐qPCR analysis of *hpo* and *CG15120* transcript levels following emCRISPRi targeting of *hpo*. Each data point in the bar graph represents an independent biological replicate, derived from the average of two technical replicates. Data are presented as mean ± SD (n = 4 biological replicates). Statistical significance was determined using two‐tailed Student's *t*‐test in GraphPad Prism. ****, *p* < 0.0001; ns indicates no significance.

Bidirectional promoters, which may regulate the co‐expression of two closely associated genes [70, 71, 72, 73], present unique challenges for achieving gene‐specific silencing due to the proximity of TSS. To evaluate the precision of emCRISPRi in such contexts, we analyzed three gene pairs with bidirectional promoters: *dCUL1*/*CG12159*, *Gpat4*/*yki*, and *hpo*/*CG15120*. The distance between TSSs in these pairs ranged from approximately about 350 bp to about 900 bp (Figure 4A,C,E).

Using *MS1096‐GAL4* driver to express dCas9‐MT(I), we measured the transcript levels of both genes in each pair via qRT‐PCR (Figure 4B,D,F). emCRISPRi silenced the target gene in all three pairs with no detectable effect on the expression of the adjacent, non‐targeted gene. For instance, silencing *dCUL1* reduced its mRNA levels by approximately 30% without affecting *CG12159* expression (Figure 4B). Similarly, emCRISPRi achieved over 50% silencing of *yki* and *hpo*, with no measurable impact on the expression of the adjacent genes *Gpat4* and *CG15120*, respectively (Figure 4D,F). These results demonstrate that the precision of emCRISPRi in achieving gene‐specific silencing even within complex promoter configurations, further highlighting its utility of dissecting regulatory networks in *Drosophila*.

### 2.9 Repression of Enhancer Activity Using emCRISPRi

In addition to targeting gene promoters, we evaluated the ability of emCRISPRi to repress cis‐regulatory elements (CREs), such as enhancers, which play critical roles in modulating gene expression [74]. As a model, we targeted the T1 CRE region of the *Scr* gene, a well‐characterized enhancer located approximately 35 kb upstream of *Scr*. This enhancer drives first thoracic leg (T1 leg) development and male T1 sex comb formation through transcription factor binding at En and Dll regulatory site [75, 76].

**FIGURE 5:**
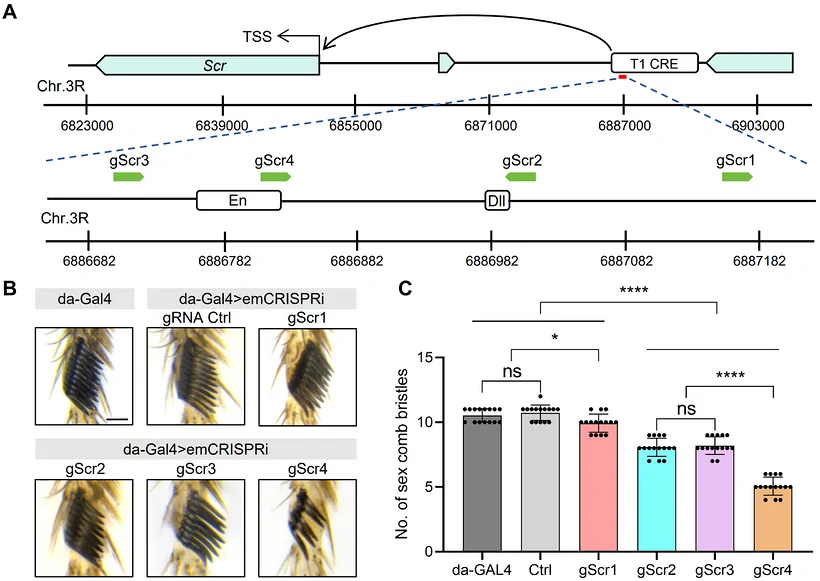
emCRISPRi effectively silences enhancer activity (A) Genomic map of the T1 cis‐regulatory element (CRE) region of the Scr gene on chromosome 3R. The positions of gRNAs (gScr1‐4) targeting the T1 CRE are shown relative to transcription factor binding sites (TSS) for En and Dll, which regulate enhancer activity. (B) Microscopic images of *Drosophila* male sex combs showing the phenotypic observation following emCRISPRi targeting of the T1 CRE region. All constructs were expressed under the control of the da‐Gal4 driver. Images are representative of biological replicates, with the specific sample size (n) for each group indicated in the panels. Scale bar: 25 µm. (gRNA Ctrl: da‐Gal4 driven emCRISPRi system with a gRNA targeting blue fluorescent protein). (C) Quantitative comparison of the number of bristles in the sex combs of male first thoracic legs for different gRNA targets. All four gRNAs (gScr1‐4) were driven by the U6:1 promoter, and emCRISPRi protein expression was controlled by *da‐Gal4*. Each data point represents an individual male T1 leg (n = 15 legs per genotype). Data are presented as mean ± SD. Statistical analysis was performed using one‐way ANOVA with Tukey's honestly significant difference (HSD) test in GraphPad Prism. *, *p* < 0.05; ****, *p* < 0.0001; ns indicates no significance.

We designed four gRNAs (gScr1, gScr2, gScr3, and gScr4) targeting the En and Dll binding sites within the T1 CRE region (Figure 5A). Flies carrying these gRNAs were crossed with those expressing emCRISPRi under the ubiquitous *da‐Gal4* driver, and T1 sex comb phenotypes were analyzed in male progeny. Among the tested gRNAs, gScr2 (targeting the Dll‐binding region) and gScr4 (targeting the En‐binding region) caused significant reductions in T1 sex comb numbers (Figure 5B,C). Flies expressing gScr2 exhibited an average of 8 sex combs per leg compared to 11 in wild‐type flies, while gScr4 reduced sex comb numbers to approximately 5 per leg. In contrast, gScr1 and gScr3, which target sites farther form the transcription factor binding regions, had weaker effects on T1 sex comb formation (Figure 5C). These results demonstrate that emCRISPRi can effectively repress enhancer activity, particularly when targeting critical transcription factor binding sites. This capability establishes emCRISPRi as powerful tool for studying enhancer function and the roles of cis‐regulatory elements in transcriptional regulation.

### 2.10 Application in Genetic Interactions Studies and Disease Modeling

**FIGURE 6:**
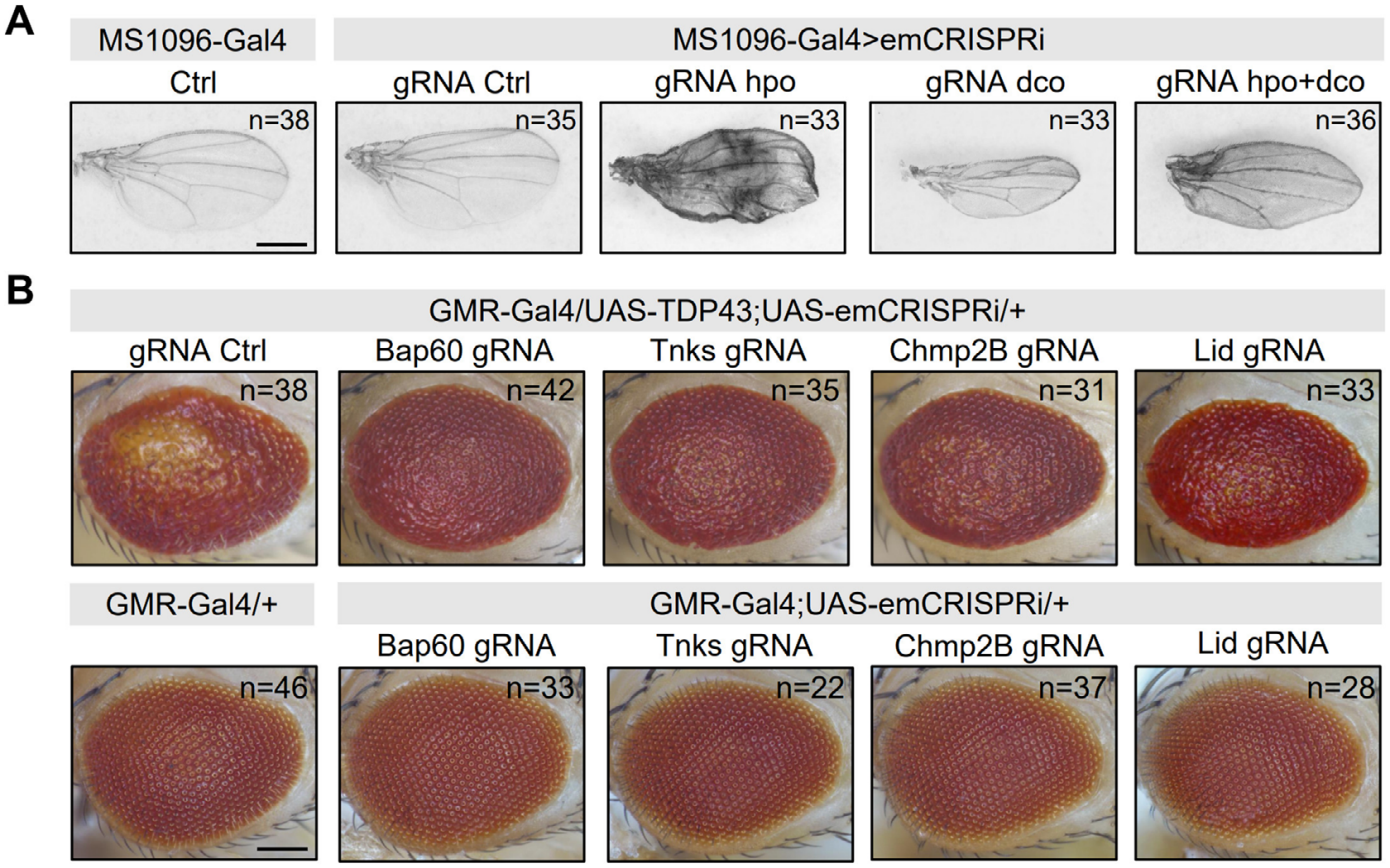
Application of emCRISPRi in genetic interactions studies and disease‐related gene screening. (A) Microscopic images of *Drosophila* adult wings showing multiplexed transcriptional inhibition of Hippo pathway components using MS1096‐Gal4. One gRNA was used per target gene. Images are representative of biological replicates, with the specific sample size (n) for each group indicated in the panels. Scale bar: 500 µm. (gRNA Ctrl: MS1096‐Gal4 driven emCRISPRi system with a gRNA targeting blue fluorescent protein). (B) Microscopic images of *Drosophila* adult eyes showing that emCRISPRi‐mediated targeting of genes regulating TDP‐43 protein toxicity mitigates the lesions induced by TDP‐43 overexpression. All constructs were driven by the GMR‐Gal4 driver. Images are representative of biological replicates, with the specific sample size (n) for each group indicated in the panels. Scale bar: 100 µm.

To explore the utility of emCRISPRi in genetic interactions, we applied it to the Hippo signaling pathway, a key regulator of cell proliferation and apoptosis during Drosophila wing development [77, 78]. Targeting *hpo* with emCRISPRi induced wing tissue overgrowth, consistent with its role as a negative regulator of growth [79] (Figure 6A; Figure S13). Conversely, silencing *dco*, a positive regulator of cell division [80], resulted in reduced wing size and formation of shrunken wings (Figure 6A; Figure S13). Notably, simultaneous silencing *hpo* and *dco* partially rescued the overgrowth phenotype caused by *hpo* silencing alone (Figure 6A; Figure S13), demonstrating the utility of emCRISPRi for probing genetic interactions within complex signaling pathways.

When targeting *warts* (*wts)*, another key gene in the Hippo pathway, neither of the two designed gRNAs (*wts‐1* and *wts‐2*) produced visible phenotypic changes (Figure S14). This result contrasts with prior studies [81], and we speculate that the lack of effect may stem from the limited targeting efficiency of the selected gRNAs.

Finally, we applied emCRISPRi to a *Drosophila* model of amyotrophic lateral sclerosis (ALS), characterized by TDP‐43 protein aggregation [82]. Overexpression of TDP‐43 in the compound eye caused retinal cytotoxicity, leading to swelling and morphological abnormalities [83]. Silencing known genetic modifiers of TDP‐43 toxicity (*Bap60*, *Tnks*, *Chmp2B*, and *Lid*) [84, 85, 86] using emCRISPRi partially rescued these phenotypes, as evidenced by reduced swelling and improved retinal morphology (Figure 6B). These results underscore the potential of emCRISPRi as a versatile tool for studying genetic interactions and modeling human diseases, offering new avenues for identifying therapeutic targets and elucidating disease mechanisms.

## 3 Discussion

In this study, we developed embedded CRISPR interference (emCRISPRi), a next‐generation gene‐silencing platform optimized for Drosophila. By embedding transcriptional repression domains (Mxi and TRD) directly within a structurally flexible region of dCas9, we achieved significantly improvements in silencing efficiency compared to conventional CRISPRi systems and RNAi. This approach addresses key limitations of existing methods, including insufficient repression and the inability to target non‐coding regulatory regions, positioning emCRISPRi as a robust and versatile tool for functional genomics, regulatory biology, and disease modeling in *Drosophila*.

Our findings demonstrate the structural and functional superiority of the embedded configuration. Structural modeling identified a flexible region within dCas9 (residues V206–D207) that allowed precise incorporation of repression domains without disrupting the protein's DNA‐binding ability. This configuration consistently outperformed conventional N‐ and C‐terminal domain fusions, achieving silencing that produced phenotypes comparable to genetics null mutants.

Interestingly, in our testing system, dCas9‐KRAB fusions failed to significantly enhance gene silencing. In higher vertebrates, the KRAB domain represses transcription by recruiting TRIM28 [87], which scaffolds heterochromatin‐inducing complexes such as nucleosome remodeling and deacetylase (NuRD) and the histone methyltransferase SETDB1 [88, 89]. In *Drosophila*, the homolog of TRIM28 is Bonus [90]; however, the KRAB‐interacting region in TRIM28 shares less than 20% sequence similarity with its counterpart in Bonus [91] (Figure S15A). This substantial divergence likely impairs KRAB‐mediated recruitment in flies. In contrast, the Mxi repression domain interacts with Sin3A, which associates with histone deacetylases to repress transcription. Notably, *Drosophila* Sin3A (dSin3A) exhibits high sequence similarity to its human counterpart (hSin3A), with over 70% similarity in the Mxi‐interacting region, including complete conservation of key residues [35] (Figure S15B). AlphaFold modeling further predicts highly conserved interaction modes between Mxi and both Sin3A homologs (Figure S15C, D,E). This strong sequence and structural conservation likely underlie the superior gene silencing efficiency of Mxi in *Drosophila*. These findings underscore the importance of tailoring CRISPRi systems to the unique molecular and regulatory landscapes of specific organisms, ensuring compatibility with endogenous transcriptional machinery.

A major strength of emCRISPRi lies in its ability to achieve robust transcriptional silencing without inducing DNA double‐strand breaks (DSBs). Traditional CRISPR systems rely on DSBs, which often introduce genomic instability through imprecise repair mechanisms [92]. In contrast, emCRISPRi operates solely at the transcriptional level, ensuring high specificity while avoiding unintended mutations or indels. This feature makes emCRISPRi particularly valuable for studying essential genes, where generating null mutants is impractical, and for achieving conditional silencing in specific tissues or developmental stages.

Direct comparisons with RNAi further highlight the advantages of emCRISPRi. While RNAi is widely used in Drosophila, it is often limited by incomplete knockdown, off‐target effects, and requirement for modified rescue constructs to prevent mRNA degradation [14]. In contrast, emCRISPRi achieved more consistent and efficient knockdown across a wide range of genes, including those with diverse promoter architectures, and allowed for functional rescue using unmodified cDNA. These practical advantages simplify experimental workflows and improve reproducibility, making emCRISPRi an ideal platform for high‐throughput genetic screens and detailed functional studies.

The enhanced silencing efficiency of CRISPRi relative to RNAi has been well‐documented in previous studies. For example, Gilbert et al. demonstrated that CRISPRi consistently achieved stronger transcriptional repression than RNAi across dozens of genomic loci [39]. Similarly, Zheng et al. reported that CRISPRi outperformed RNAi in silencing multiple genes in primary neuronal cells [93]. Our findings further extend these observations to *Drosophila*, where emCRISPRi demonstrated superior knockdown efficiency and consistency compared to RNAi.

In addition to enhanced efficacy, CRISPRi provides greater targeting precision relative to RNAi. Gilbert et al. showed that CRISPRi induces significantly fewer off‐target effects than RNAi [39], and Evers et al. demonstrated that CRISPR‐based genetic screens produce more reliable essential‐gene sets than shRNA screens, which suffer from higher noise due to off‐target activity [10]. Mechanistically, CRISPRi's specificity arises from the dual requirement of gRNA complementarity and the presence of a nearby PAM sequence, a more stringent condition than the seed‐based matching that drives many RNAi off‐target effects [1]. Additionally, CRISPRi's reliance on TSS‐proximal targeting reduces the likelihood of unintended regulatory interactions, further enhancing its precision [39, 94]. Together, these findings establish emCRISPRi as a superior alternative to RNAi, combining high efficiency with unparalleled specificity.

Another notable feature of CRISPRi is its strand‐independent mechanism of action. Previous studies in eukaryotic cells have demonstrated that targeting either the template or the non‐template strand does not significantly impact repression efficiency [39, 94]. This is because the dCas9 complex acts as a physical roadblock to RNA polymerase elongation, effectively repressing transcription regardless of the strand it binds to.

Despite its advantages, the high targeting precision of CRISPRi necessitates rigorous gRNA design. CRISPRi‐mediated repression can be abolished by PAM alterations or the presence of two or more mismatches between the gRNA and the target sequence [39]. Even a single‐base mismatch in the seed region can severely disrupt binding and impair silencing [93]. While this sensitivity minimizes off‐target effects, it also introduces the potential for single nucleotide polymorphisms (SNPs) to allow escape from silencing. Therefore, comprehensive in silico analysis is critical for prioritizing gRNAs with minimal off‐target potential and ensuring target sequence conservation across genetic backgrounds [95, 96, 97]. Beyond sequence complementarity, the 5' terminal composition of the gRNA is critical for silencing efficacy. While bioinformatics tools often identify gRNA that do not initiate with a purine (G), effective in vivo expression under the U6 promoter, which preferentially initiates transcription with a ‘G,’ requires appending an extra guanine at the 5' end. This modification improves U6‐driven transcription efficiency and reduces the likelihood of suboptimal gene silencing due to insufficient gRNA abundance [98]. Validation of key targets using methods such as qRT‐PCR or RNA‐Seq is strongly recommended to confirm specificity and efficacy.

Beyond classical gene silencing, emCRISPRi demonstrated exceptional versatility in targeting cis‐regulatory elements (CREs), such as enhancers. By targeting transcription factor binding sites within enhancers, emCRISPRi achieved precise and effective repression, revealing dependencies on gRNA proximity to key regulatory regions. These findings expand the utility of emCRISPRi beyond promoter regions, addressing limitations of RNAi and RNA‐targeting CRISPR systems (e.g., RfxCas13d [16]) in studying non‐coding regulatory sequences. This capability establishes a framework for dissecting enhancer function and exploring the regulatory mechanisms underlying gene expression in tissue‐specific contexts.

To provide a comprehensive overview of the distinctions among gene‐silencing platforms, we have included a summary table comparing key features of RNAi, first‐generation CRISPRi, emCRISPRi and CRISPR/Cas13 system (Table S1). This table highlights differences in silencing mechanisms, silencing efficiency, ability to target non‐coding regulatory elements, off‐target considerations, and capacity for phenotypic rescue. Such comparisons offer a concise reference for researchers seeking to evaluate the practical advantages and limitations of each system in different experimental context.

Additionally, emCRISPRi proved effective in silencing multiple genes simultaneously, enabling the study of complex genetic interactions. For example, in the Hippo signaling pathway, emCRISPRi revealed compensatory mechanisms in growth regulation by simultaneously silencing *hpo* and *dco*. Furthermore, its utility was demonstrated in a *Drosophila* model of amyotrophic lateral sclerosis (ALS), where silencing genetic modifiers of TDP‐43 toxicity mitigated disease phenotypes. These findings underscore the potential of emCRISPRi for identifying therapeutic targets and advancing our understanding multifactorial diseases.

Another important consideration is the heterogeneous activity of the *MS1096‐Gal4* driver used in our wing assays. This driver exhibits strong expression in the dorsal compartment of the wing disc but weaker or mosaic activity in the ventral and proximal regions [53, 54]. Consequently, the residual mRNA levels detected in whole‐tissue qPCR likely reflect contributions from cells with low or absent Gal4 expression rather than limitations of the emCRISPRi system itself. These findings highlight the critical role of driver‐specific expression patterns in interpreting silencing results, particularly in spatially heterogeneous tissues. Future studies employing alternative drivers or tissue‐specific reporters could provide a more precise assessment of emCRISPRi efficacy in different cellular contexts. In addition to these extrinsic factors, intrinsic properties of the CRISPR system also influence silencing efficiency. SpCas9 exhibits sequence preferences that can lead to variability in gRNA activity across different targets [99, 100]. Furthermore, chromatin structure plays a significant role in determining accessibility; nucleosomes and DNA‐binding proteins can sterically hinder dCas9 binding at certain loci, reducing targeting efficiency [101]. These factors suggest that gRNA design must consider not only sequence complementarity and proximity to the TSS but also the chromatin landscape of the target region. Advanced computational tools and chromatin accessibility data could be integrated into gRNA selection pipelines to enhance targeting success.

While Mxi and TRD were effective in this study, exploring alternative repression domains or combinations could further enhance silencing efficiency. The inherent flexibility of the embedded design also presents opportunities to incorporate activation domains, facilitating the development of CRISPR activation (CRISPRa) systems for genome‐wide overexpression studies [39, 102, 103, 104].

Although this study focused on *Drosophila*, the embedded design has significant potential for broader applications in other model organisms and therapeutic contexts. In mammalian systems, embedding repression domains directly within dCas9 could enhance silencing efficiency and overcome limitations of existing CRISPRi systems. Notably, embedded fusion strategies have already been adopted in base editors and prime editors, where inserting enzymatic domains into internal regions of Cas9 has improved editing precision and expanded targeting scope [27, 28, 29, 30, 31, 105, 106, 107], demonstrating the broader feasibility of this internal fusion approach for advanced genome engineering applications. Applying similar strategies to CRISPRi could further improve silencing efficiency and targeting precision, particularly in therapeutic settings where precise control of gene expression is critical.

In conclusion, emCRISPRi represents a transformative advancement in conditional gene‐silencing technology. By embedding transcriptional repression domains within dCas9, this system achieves unparalleled efficiency and versatility in Drosophila. Its ability to target both coding and non‐coding regions, combined with its compatibility with unmodified cDNA rescue, establishes emCRISPRi as a powerful platform for functional genomics, enhancer biology, and disease modeling. With further optimization and adaptation, emCRISPRi holds immense potential for advancing genetic research across diverse biological systems and contributing to the development of novel therapeutic strategies.

## 4 Methods and Materials

### 4.1 Tool Flies

The following *Drosophila melanogaster* tool strains were used in this study: *GMR‐Gal4*, *vasa‐Gal4*, *ey‐Gal4*, *MS1096‐Gal4*, *da‐Gal4*, and *Act5C‐Gal4*. These strains are maintained and deposited in our laboratory. The ALS disease model flies (*UAS‐TDP43‐YEP/cyo; GMR‐Gal4 (YH3) TM6B*) used in this study were generously provided by Dr. Yanshan Fang from the Interdisciplinary Research Center on Biology and Chemistry, Shanghai Institute of Organic Chemistry, Chinese Academy of Sciences, Shanghai, China, as originally described in Elden et al. [108].

### 4.2 RNAi Experiments

All RNAi *Drosophila* lines used in this study were obtained from the Tsinghua Fly Center (Beijing, China). Detailed information regarding these lines, including stock numbers and target genes, is provided in Table S2. Following crosses between RNAi and GAL4 lines, the progenies were maintained at 27 ± 0.5°C with 60 ± 5% relative humidity under a 12 h light/12 h dark photoperiod.

### 4.3 Plasmid Construction

#### 4.3.1 CRISPRi Plasmid Backbones

The backbone of the CRISPRi plasmids was constructed through modular assembly of several functional components. The ampicillin resistance gene (AmpR) and the origin of replication (ori) for prokaryotic selection and propagation were derived from the pUC19 plasmid [109]. The regulatory cassette consisting of phiC31 attB‐gypsy‐10UAS‐Hsp70‐Ftz intron‐SV40 polyA‐gypsy was adapted from the pWalium20 vector with minor sequence modifications [110]. The gRNA scaffold was obtained from the pCFD4 plasmid (Addgene #49411) [98], and the 3P3‐RFP‐Tub‐UTR marker system was sourced from the pRFP‐U6B/CR7T‐gRNA plasmid [111]. These four components were assembled by molecular cloning into a pre‐CRISPRi backbone.

The dSpCas9 coding sequence was derived from the pGTL‐112 plasmid [11]. The Mxi and MeCP2‐TRD domains, both codon‐optimized for *Drosophila* expression, were chemically synthesized by GenScript (Suzhou, China). The KRAB domain was obtained from the pHR‐SFFV‐dCas9‐BFP‐KRAB plasmid (Addgene #46911) [32]. These repressor domains were incorporated into the CRISPRi backbone via different fusion strategies and cloned through pre‐engineered NotI and NheI restriction sites, generating the series of CRISPRi plasmid backbones used in this study. The plasmid sequence of the final version of the emCRISPRi system is provided in Table S3.

### 4.4 Construction of Functional CRISPRi Knockdown Plasmids

gRNAs targeting each gene were designed using CRISPRscan [96] and individually cloned into the CRISPRi backbones using BsaI or BsmBI (New England Biolabs, NEB) followed by ligation with T4 DNA ligase (NEB). Each knockdown plasmid was engineered to contain one or two gRNAs, expressed under either the U6:1 or U6:3 promoter. For constructs expressing a single gRNA, the U6:1 promoter was consistently used for its expression, while the second cassette under the U6:3 promoter contained a placeholder sequence (GGAGACCATGCGGTCTCC) including two BsaI sites. The gRNA sequences used in this study are listed in Table S4.

### 4.5 Construction of Rescue cDNA Plasmids

For cDNA rescue experiments, the white gene in the pWalium20 plasmid [110] was replaced with the 3P3‐GFP‐Tublin polyA cassette derived from PiggyBac‐10UAS‐Cas9 [111] using NsiI and XhoI, generating the pW20‐EGFP vector. The target cDNA was then inserted downstream of the 10×UAS‐Hsp70 promoter via NheI digestion followed by Gibson Assembly (New England Biolabs, NEB), yielding the final rescue plasmids. The plasmid sequence of pW20‐EGFP is provided in Table S3.

### 4.6 Acquisition of Unmodified cDNA

Total RNA was extracted from *Drosophila* larvae using TRIzol reagent. Subsequently, reverse transcription was performed with the PrimeScript RT Master Mix (Takara, RR036A) to obtain cDNA. The unmodified cDNA was then amplified via PCR using gene‐specific primers. The primer sequences used in this study are provided in Table S5.

### 4.7 Generation of Transgenic Lines (CRISPRi, and cDNA Rescue)

All transgenic *Drosophila* lines were generated through embryo microinjection. The procedure utilized attP40 embryos, as previously described in the literature [112]. Briefly, newly enclosed attp40 fruit flies were mated in culture vials at a ratio of 150 males to 150 females and provided with fresh yeast paste as a food source. Embryos were collected at 40‐min intervals, dechorionated under a stereomicroscope, and aligned on double‐sided tape attached to glass slides. Following dechorionated, embryos were dried in a desiccator for 5 min and coated with halocarbon oil to prevent desiccation. The slides were then transferred to an embryo microinjection system equipped with a stereomicroscope, and the plasmid solution was injected into the posterior pole of each embryo. After injection, the slides were placed in a humidified chamber and incubated at 19°C for 36 ∼ 40 h to allow proper development. Healthy larvae were then transferred to standard food vials and maintained at 25°C for further development. Once the microinjected larvae enclosed into adult flies, they were mated with newly enclosed *w*1118 *Drosophila* at a 1:3 ratio in a 25°C constant temperature incubator. Transgenic flies were identified by screening for RFP or GFP fluorescence under a fluorescence stereomicroscope.

### 4.8 Microscopy Imaging

*Drosophila* in culture vials were subjected to CO_2_ anesthesia, after which the immobilized flies were transferred to EP tubes placed on ice to sustain the anesthetized state. Subsequently, the anesthetized flies were carefully repositioned on the stage plate of a Nikon SMZ‐25 macro zoom stereomicroscope. Adjustments were made to the positioning or dissection of the flies as required for optimal image acquisition. The acquired images were processed using NIS‐Elements Viewer 5.21 software for initial analysis.

### 4.9 RNA Extraction, cDNA Synthesis and RT‐ qPCR Assay

For each biological replicate, total RNA was extracted from 20 pairs wing discs, eye‐disc or adult flies with TRIzol reagent, following the manufacturer's instructions. The extracted RNA was treated with DNase to remove any residual genomic DNA. RNA samples were reverse‐transcribed using PrimeScript RT Master Mix (Takara, RR036A), with 1 µg of RNA used for each reaction. The cDNA obtained was used for the subsequent RT‐qPCR experiments. Real‐time quantitative PCR was performed using the Hieff qPCR SYBR Green Master Mix (Rox Provided Separately) kit (Yeasen, #11204ES08) on a QuantStudio 7 Flex Real‐Time PCR system (Life Technologies). The expression levels of target genes were quantified using the comparative CT method, with rp49 serving as the endogenous reference gene. Each experiment included at least three independent biological replicates. For each biological replicate, two technical replicates were performed and their values were averaged before statistical analysis. The primer sequences used in this study are provided in Table S6. All primers were synthesized by Ruimian (Shanghai) and verified for specificity and efficiency prior to use.

### 4.10 Assessment of Progeny Viability

Male flies with the genotype *dCas9‐MT(I)‐RFP/cyo* were crossed with homozygous virgin female flies of the *Actin‐Gal4* strain. The cultures were maintained at both 27°C and 29°C, with 10 parallel repeats for each condition to ensure statistical reliability. According to Mendelian principles of segregation and independent assortment, in the absence of lethality, adult offspring would be expected to display a roughly equal proportion of *Actin‐Gal4/ dCas9‐MT(I)‐RFP* and *Actin‐Gal4/cyo* phenotypes. However, if lethality occurs in the offspring, this expected phenotypic ratio would be disrupted. Statistical analysis of the offspring revealed that the observed ratios were consistent with the expected Mendelian ratios in the absence of lethality.

### 4.11 Statistical Analysis

Statistical analyses were performed using GraphPad Prism 8.0 software. The exact sample sizes (n) for each experimental group are indicated in the corresponding figure. In qPCR experiments, samples were normalized to the housekeeping gene rp49 using the ΔΔCT method [113]. For comparisons between two groups, a two‐tailed Student's t‐test was used. For multiple pairwise comparisons, one‐way ANOVA with Tukey's honestly significant difference (HSD) test was applied. Data in figures are presented as mean ± SD of these biological replicates. Statistical significance is indicated as follows: * *p* < 0.05, ** *p* < 0.01, *** *p* < 0.001, and **** *p* < 0.0001; ‘ns’ denotes no significance.

### 4.12 Preservation and Culture of Flies

Transgenic and tool fly strains were maintained under standard laboratory conditions at 19 ± 0.5°C, 60 ± 5% relative humidity, with a 12 h light/12 h dark photoperiod. To minimize environmental influences on phenotypic traits, progeny derived from crosses between these strains were reared under controlled conditions of 27 ± 0.5°C, 60 ± 5% relative humidity, with a 12 h light/12 h dark photoperiod. All experiments were performed using synchronized fly populations, and larvae were cultured at defined densities to reduce variability in developmental and physiological parameters.

### 4.13 Illustration

Generated by employing Microsoft PowerPoint and Biorender (www.biorender.com).

## Author Contributions

G.G. and Z.X. designed and supervised the whole project. Z.X., F.P., Z.Y., Z.K. and S.A. generated the transgenic flies. Z.X., F.P. and Z.Y. performed the imaging collection and analysis. Z.X., F.P., Z.J. and B.W. performed molecular experiments. F.P., Z.X., and G.G. wrote the manuscript. All authors discussed the results and commented on the manuscript.

## Conflicts of Interest

The authors declare no conflict of interest.

## Supporting information




**Supporting File 1**: advs74922‐sup‐0001‐SuppMat.pdf.

**Supporting File 2**: advs74922‐sup‐0002‐Table1.xlsx.

## Data Availability

The data that support the findings of this study are available in the supplementary material of this article.
