# Supplementary material for: Embedded CRISPRi Enhances Gene‐Silencing Efficiency in Drosophila
Source: Adv Sci (Weinh). 2026 Mar 20;13(31):e15849. doi: 10.1002/advs.202515849 (PMC13588077; doi:10.1002/advs.202515849)
Supplement: Supplementary file 1 [file ADVS-13-e15849-s001.pdf]

Supplementary information for

Embedded CRISPRi Enhances Gene-Silencing Efficiency in *Drosophila*

This PDF file includes:

Figure S1. The structure of SpCas9 and the location of domain fusion.
Figure S2. Flowchart of GAL4 system driving CRISPRi system in *Drosophila*.
Figure S3. Superior gene silencing by the V206-D207 embedded configuration.
Figure S4. RT-qPCR shows dCas9-MT(I) has higher gene-silencing efficiency.
Figure S5. KRAB is not effective in the *Drosophila* system.
Figure S6. dSpCas9-MT(I) outperforms other constructs in silencing *hpo* gene.
Figure S7. dSpCas9-MT(I) effectively silences the *e* and *y* genes.
Figure S8. Analysis of dSpCas9-MT(I) fusion protein and eclosion viability test.
Figure S9. emCRISPRi induces significant phenotypic defects in *Drosophila* wings
Figure S10. Both RNAi and emCRISPRi can induce significant phenotypic defects in *Drosophila* wings.
Figure S11. Schematic of the cDNA rescue experiment for emCRISPRi.
Figure S12. emCRISPRi can achieve transcriptional repression of various types of promoters.
Figure S13. Application of emCRISPRi system in signal pathways.
Figure S14. The wts-1 and wts-2 gRNAs failed to produce obvious phenotypic defects.
Figure S15. Sequence and structure alignment of TRIM28 and Sin3A homologues.

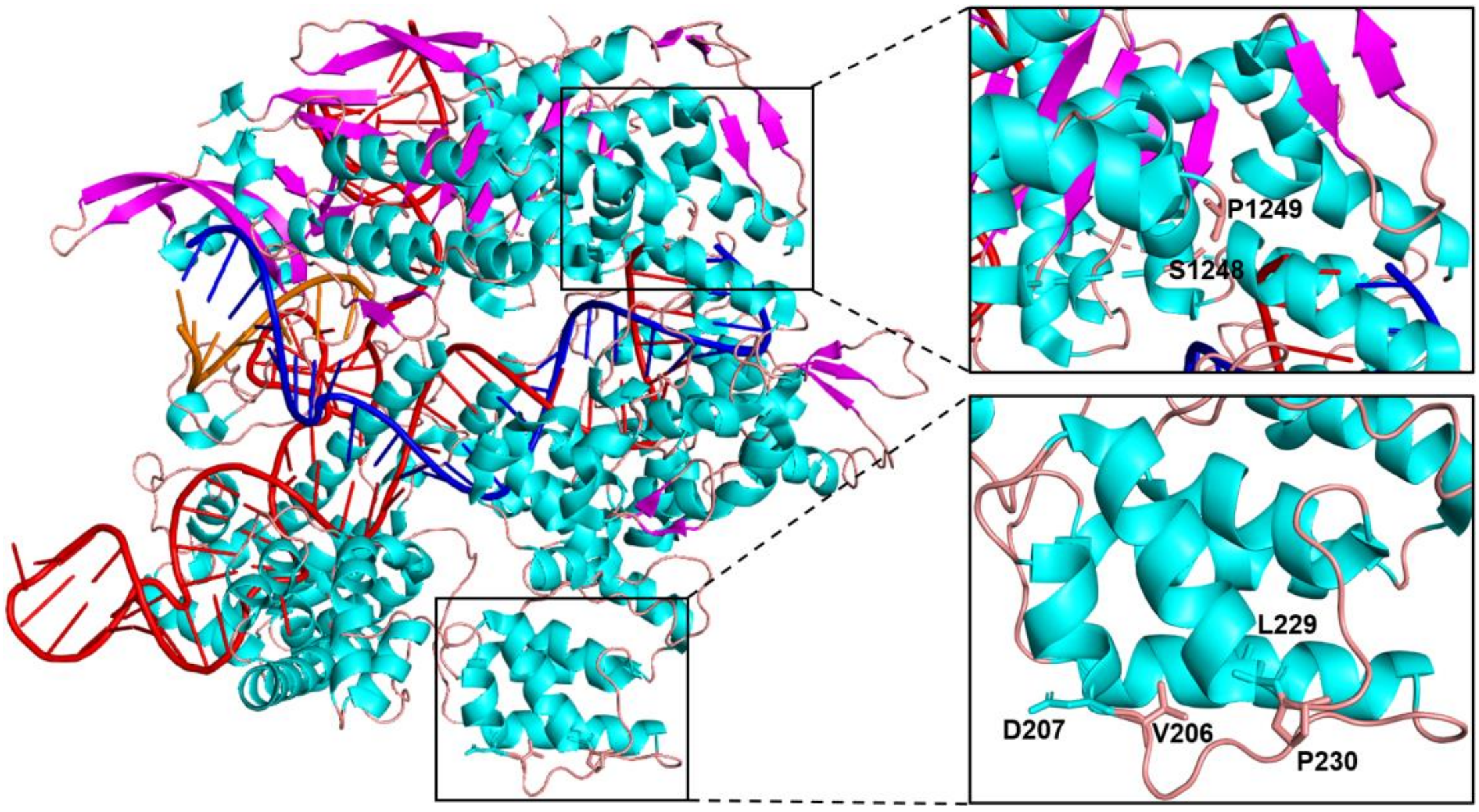

Figure S1. The structure of SpCas9 and the location of domain fusion

Structure of the SpCas9-gRNA-DNA complex (PDB: 6ai6). Amino acid residues labeled in the figure are those on both sides of the domain insertion site.

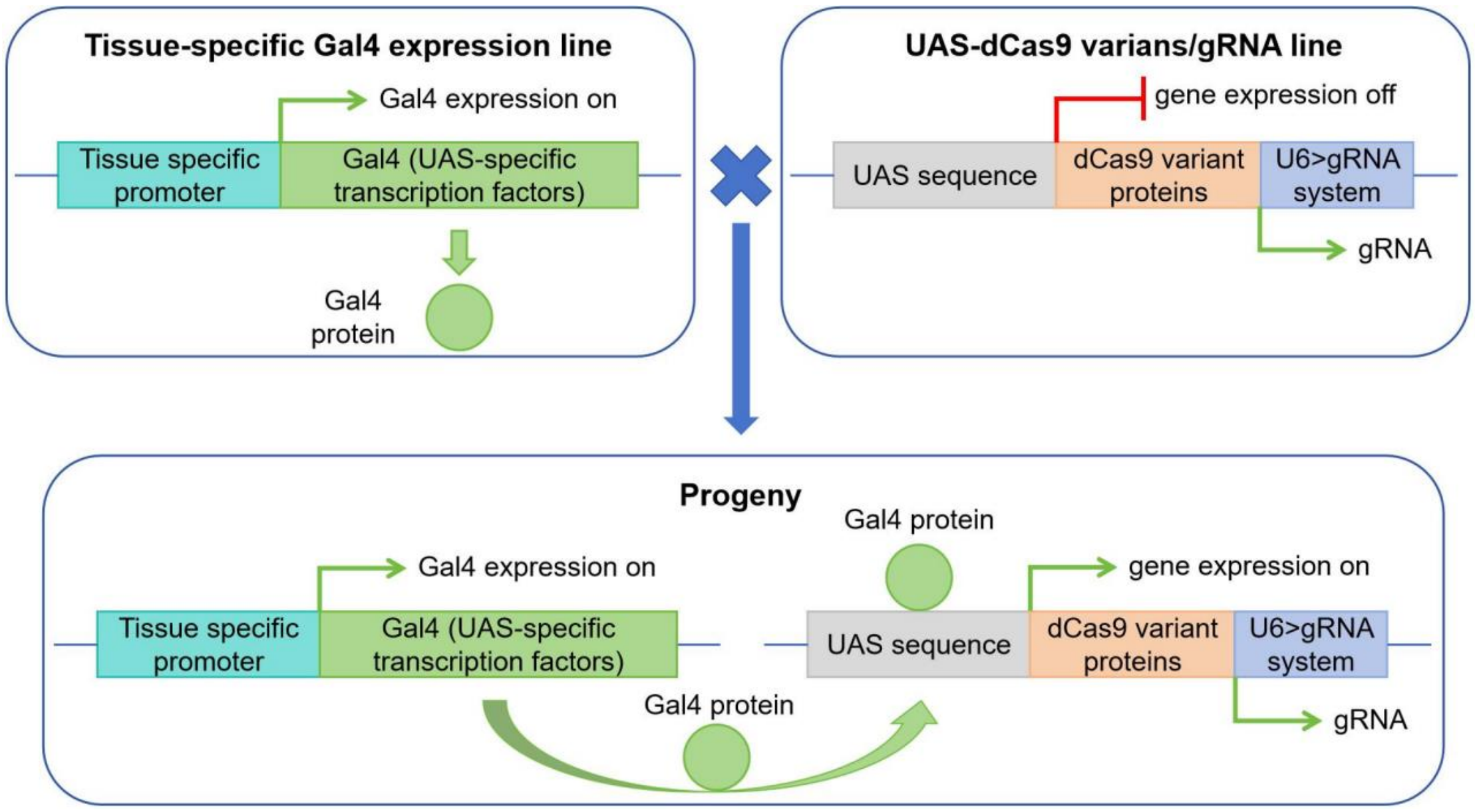

Figure S2. Flowchart of GAL4 system driving CRISPRi system in *Drosophila*

In the absence of GAL4, only the gRNA is stably expressed in the CRISPRi system, while the CRISPRi protein is not produced. When CRISPRi transgenic flies are crossed with a GAL4 driver line, progeny inheriting both systems express the CRISPRi protein under GAL4 control, enabling it to cooperate with the constitutive gRNA to transcriptionally suppress the target gene.

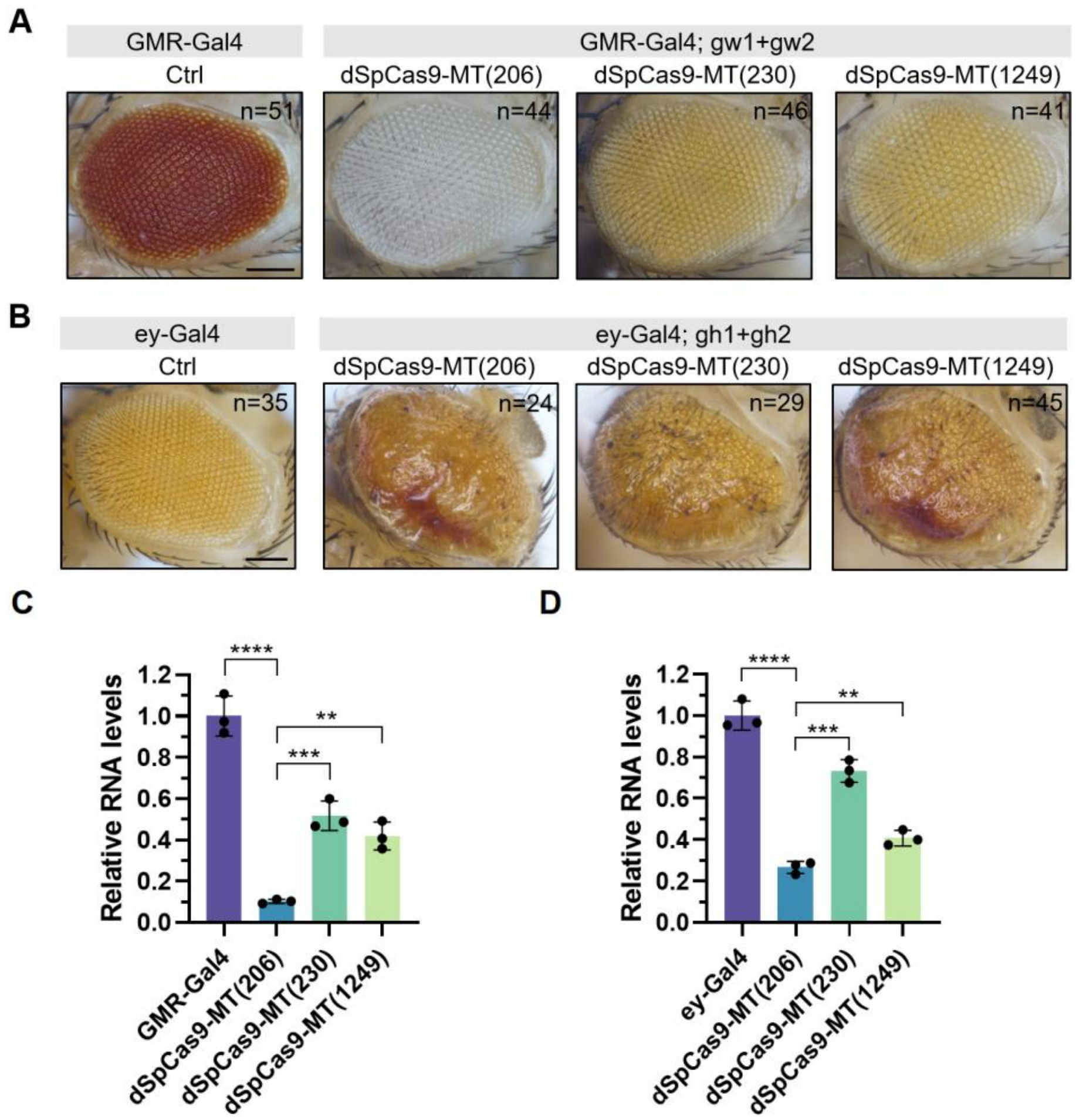

Figure S3. Superior gene silencing by the V206-D207 embedded configuration.

A) Microscopic images of *Drosophila* adult eyes showing the inhibitory effects of CRISPRi protein variants. The proteins were expressed under the control of the GMR-Gal4 driver, and the gRNAs (gw1 and gw2) were driven by the U6:1 and U6:3 promoters, respectively. Images are representative of biological replicates, with the specific sample size (n) for each group indicated in the corresponding panels. Scale bar: 100 μm. (Ctrl: *GMR-Gal4/+*).

B) Microscopic images of *Drosophila* adult eyes showing the inhibitory effects of CRISPRi protein variants. The proteins were expressed under the control of the ey-Gal4 driver, and the gRNAs (gh1 and gh2) were driven by the U6:1 and U6:3 promoters, respectively. Images are representative of biological replicates, with the specific sample size (n) for each group indicated in the corresponding panels. n = number of samples. Scale bar: 100 μm. (Ctrl: *ey-Gal4/+*).

C) Quantitative RT-qPCR analysis of *w* transcript levels under GMR-Gal4 control. The housekeeping gene *rp49* was used as an internal reference for normalization. Each data point in the bar graph represents an independent biological replicate, derived from the average of two technical replicates. Data are presented as mean ± SD (n = 3 biological replicates). Statistical analysis was performed using one-way ANOVA with Tukey's honestly significant difference (HSD) test in GraphPad Prism. *, **, *** and **** indicate $P < 0.05$, $P < 0.01$, $P < 0.001$ and $P < 0.0001$, respectively, and ns indicates no significance.

D) Quantitative RT-qPCR analysis of *hpo* transcript levels under ey-Gal4 control. The housekeeping gene *rp49* was used as an internal reference for normalization. Each data point in the bar graph represents an independent biological replicate, derived from the average of two technical replicates. Data are presented as mean ± SD (n = 3 biological replicates). Statistical analysis was performed using one-way ANOVA with Tukey's honestly significant difference (HSD) test in GraphPad Prism. *, **, *** and **** indicate $P < 0.05$, $P < 0.01$, $P < 0.001$ and $P < 0.0001$, respectively, and ns indicates no significance.

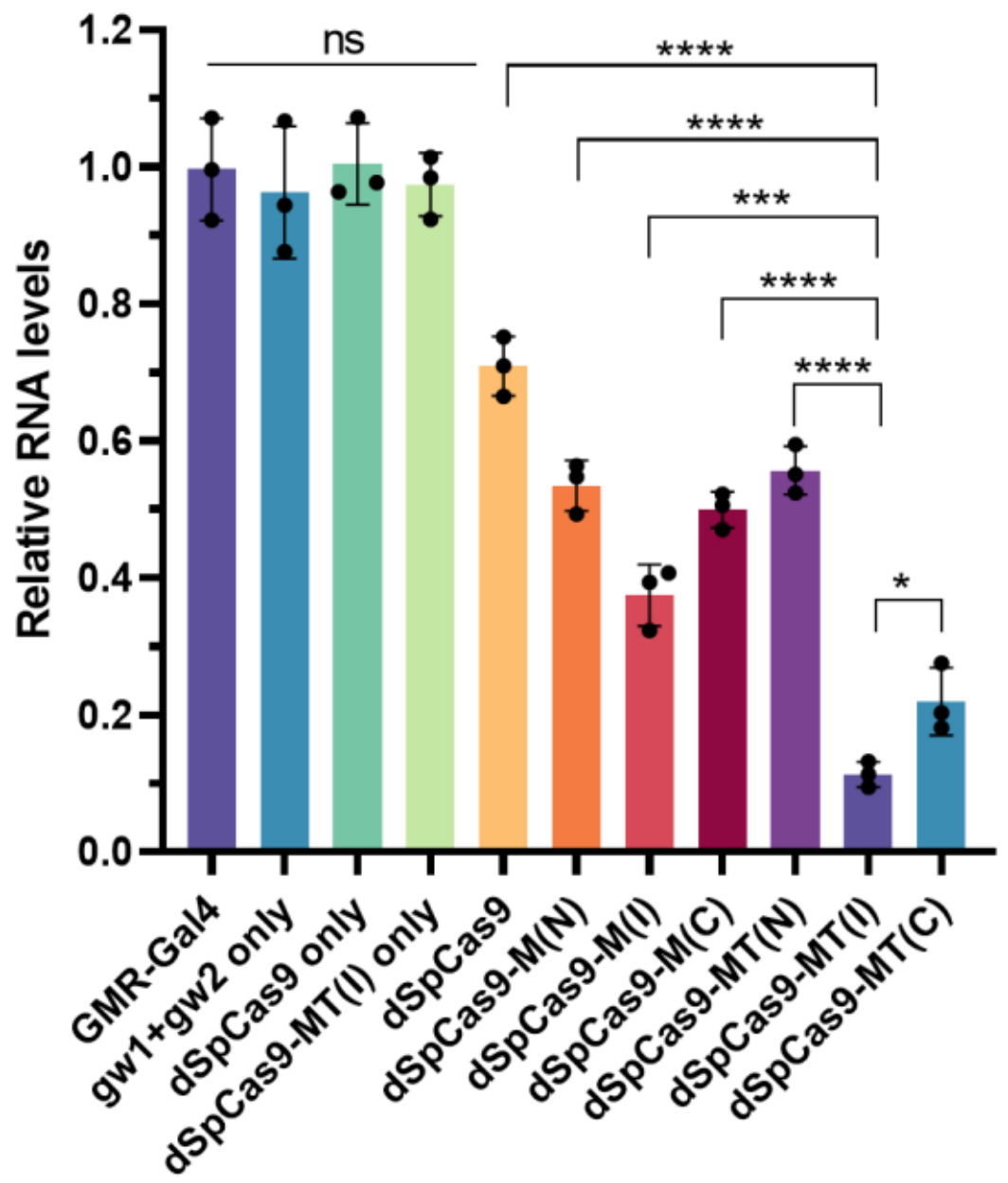

Figure S4. RT-qPCR shows dCas9-MT(I) has higher gene-silencing efficiency

Quantitative RT-qPCR analysis of *w* transcript levels under GMR-Gal4 control in Figure 1b. The housekeeping gene *rp49* was used as an internal reference for normalization. Each data point in the bar graph represents an independent biological replicate, derived from the average of two technical replicates. Data are presented as mean ± SD (n = 3 biological replicates). Statistical analysis was performed using one-way ANOVA with Tukey's honestly significant difference (HSD) test in GraphPad Prism. *, **, *** and **** indicate $P < 0.05$, $P < 0.01$, $P < 0.001$ and $P < 0.0001$, respectively, and ns indicates no significance.

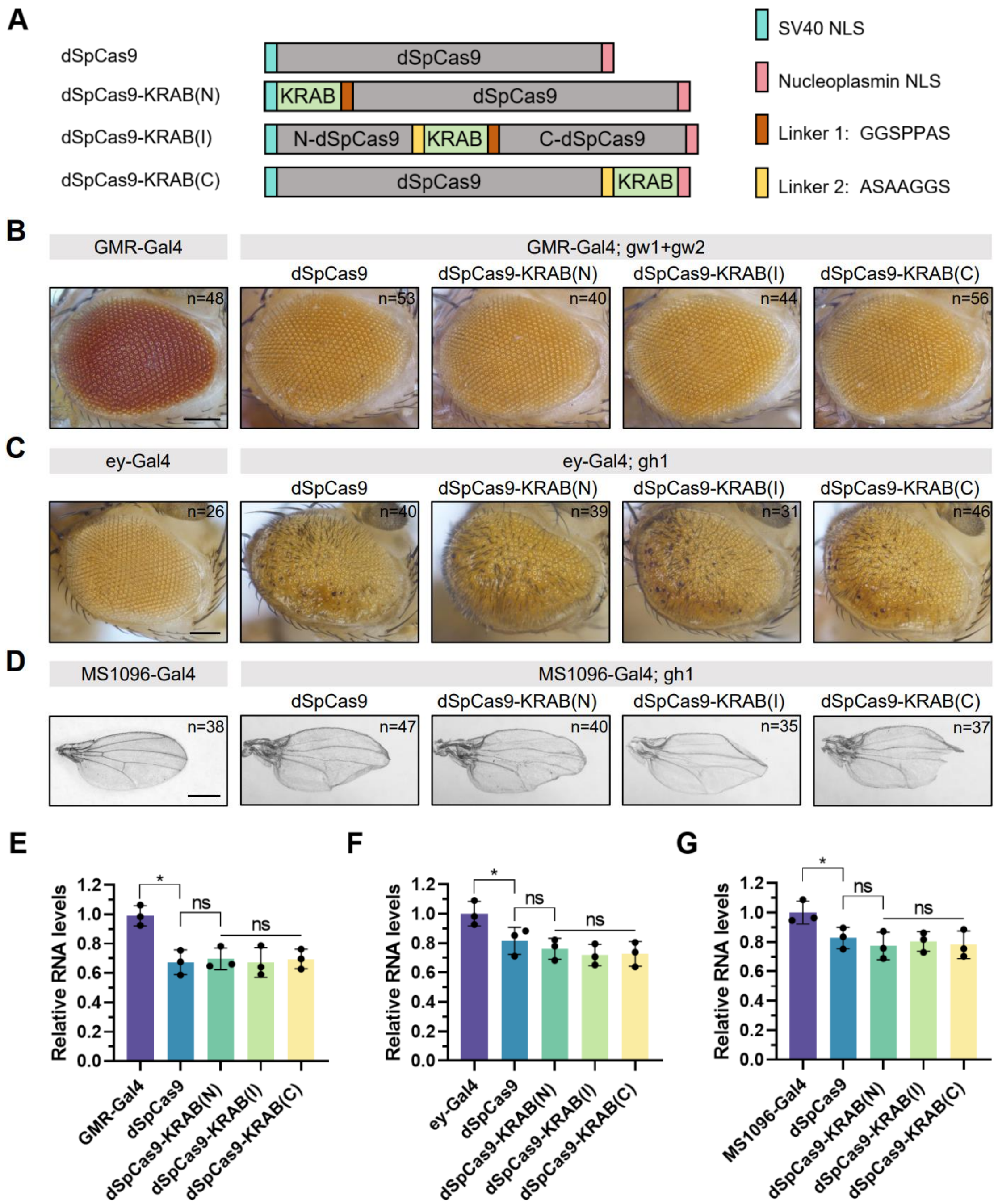

Figure S5. KRAB is not effective in the *Drosophila* system

A) Schematic representation of CRISPRi protein architectures. KRAB domains are fused to the N-terminus, embedded in the middle, and attached to the C-terminus of the dSpCas9 protein. Color coding indicates specific elements: Cornflower Blue (SV40 NLS), Scarlet (Nucleoplasmin NLS), Brown (Linker 1), Yellow (Linker 2), Gray (dSpCas9, Drosophila melanogaster codon-optimized), and Light Green (KRAB).

B) Microscopic images of *Drosophila* adult eyes showing the inhibitory effects of CRISPRi protein variants. The proteins were expressed under the control of the GMR-Gal4 driver, and the gRNAs (gw1 and gw2) were driven by the U6:1 and U6:3 promoters, respectively. Images are representative of biological replicates, with the specific

sample size (n) for each group indicated in the corresponding panels. Scale bar, 100 μm. (Ctrl is *GMR-Gal4/+*)

C) Microscopic images of *Drosophila* adult eyes showing the inhibitory effects of CRISPRi protein variants. The proteins were expressed under the control of the ey-Gal4 driver, and the gRNAs (gh1 and gh2) were driven by the U6:1 and U6:3 promoters, respectively. Images are representative of biological replicates, with the specific sample size (n) for each group indicated in the corresponding panels. Scale bar, 100 μm. (Ctrl is *ey-Gal4/+*)

D) Microscopic images of *Drosophila* adult wings showing the inhibitory effects of CRISPRi protein variants. The proteins were expressed under the control of the MS1096-Gal4 driver, and the gRNAs (gh1 and gh2) were driven by the U6:1 and U6:3 promoters, respectively. Images are representative of biological replicates, with the specific sample size (n) for each group indicated in the corresponding panels. Scale bar, 500 μm. (Ctrl is *MA1096-Gal4/+*)

E) Quantitative RT-qPCR analysis of w transcript levels under GMR-Gal4 control. The housekeeping gene *rp49* was used as an internal reference for normalization. Each data point in the bar graph represents an independent biological replicate, derived from the average of two technical replicates. Data are presented as mean ± SD (n = 3 biological replicates). Statistical analysis was performed using one-way ANOVA with Tukey's honestly significant difference (HSD) test in GraphPad Prism. *, **, *** and **** indicate $P < 0.05$, $P < 0.01$, $P < 0.001$ and $P < 0.0001$, respectively, and ns indicates no significance.

F) Quantitative RT-qPCR analysis of *hpo* transcript levels under ey-Gal4 control. The housekeeping gene *rp49* was used as an internal reference for normalization. Each data point in the bar graph represents an independent biological replicate, derived from the average of two technical replicates. Data are presented as mean ± SD (n = 3 biological replicates). Statistical analysis was performed using one-way ANOVA with Tukey's honestly significant difference (HSD) test in GraphPad Prism. *, **, *** and **** indicate $P < 0.05$, $P < 0.01$, $P < 0.001$ and $P < 0.0001$, respectively, and ns indicates no significance.

G) Quantitative RT-qPCR analysis of *hpo* transcript levels under *MS1096-Gal4* control. The housekeeping gene *rp49* was used as an internal reference for normalization. Each data point in the bar graph represents an independent biological replicate, derived from the average of two technical replicates. Data are presented as mean ± SD (n = 3 biological replicates). Statistical analysis was performed using one-way ANOVA with Tukey's honestly significant difference (HSD) test in GraphPad Prism. *, **, *** and **** indicate $P < 0.05$, $P < 0.01$, $P < 0.001$ and $P < 0.0001$, respectively, and ns indicates no significance.

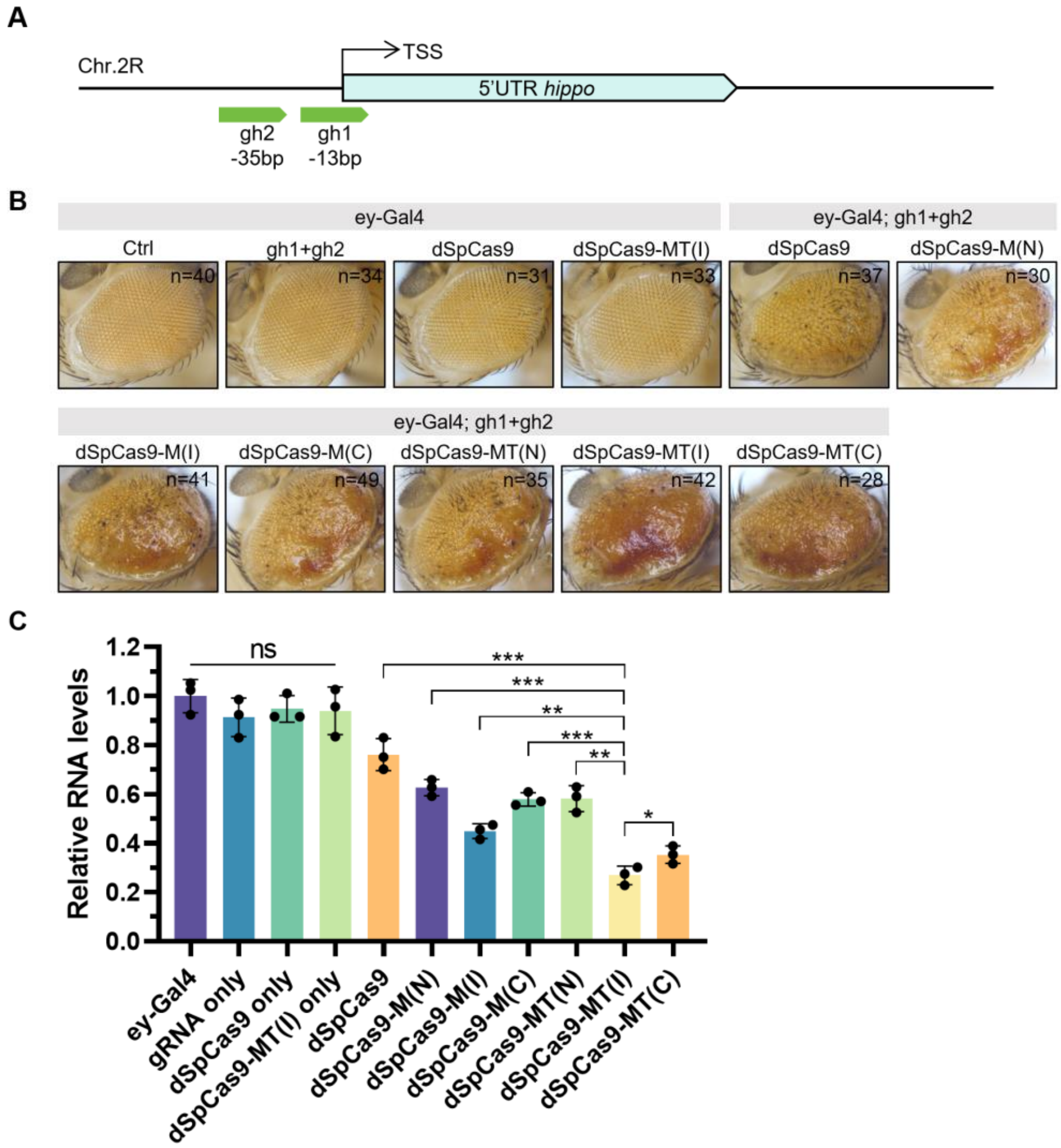

Figure S6. dSpCas9-MT(I) outperforms other constructs in silencing *hpo* gene

A) The genomic locus of the *hippo*(*hpo*) gene on the Chr.2L is shown, highlighting the positions of two designed sgRNAs (gh1 and gh2) designed near the transcription start site (TSS).

B) Microscopic images of *Drosophila* adult eyes showing the inhibitory effects of CRISPRi protein variants. The proteins were expressed under the control of the ey-Gal4 driver, and the gRNAs (gh1 and gh2) were driven by the U6:1 and U6:3 promoters, respectively. Images are representative of biological replicates, with the specific sample size (n) for each group indicated in the corresponding panels. Scale bar: 100 µm. (Ctrl: *ey-Gal4*).

C) Quantitative RT-qPCR analysis of *hpo* transcript levels under MS1096-Gal4 control. The housekeeping gene *rp49* was used as an internal reference for normalization. Each data point in the bar graph represents an independent biological replicate, derived from the average of two technical replicates. Data are presented as mean ± SD (n = 3 biological replicates). Statistical analysis was performed using one-way ANOVA with Tukey's honestly significant difference (HSD) test in GraphPad Prism. *, **, *** and **** indicate $P < 0.05$, $P < 0.01$, $P < 0.001$ and $P < 0.0001$, respectively, and ns indicates no significance.

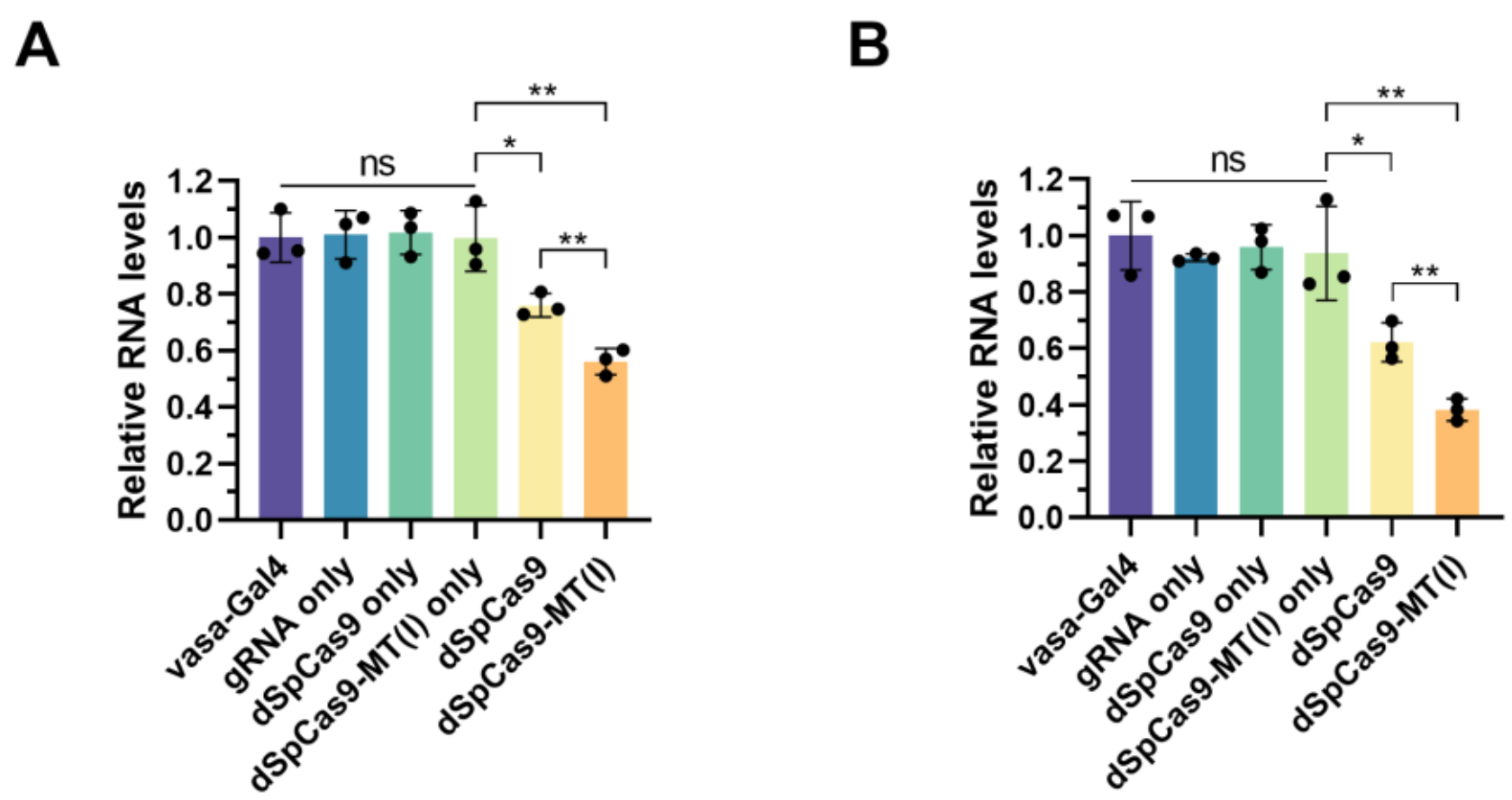

Figure S7. dSpCas9-MT(I) effectively silences the *e* and *y* genes.

Quantitative RT-qPCR analysis comparing the gene-silencing efficiency of dSpCas9 and dSpCas9-MT(I) at *e*(A) and *y*(B) gene loci. Transcript levels of target genes were measured, with rp49 serving as the internal reference for normalization. Each data point in the bar graph represents an independent biological replicate, derived from the average of two technical replicates. Data are presented as mean ± SD (n = 3 biological replicates). Statistical analysis was performed using one-way ANOVA with Tukey's honestly significant difference (HSD) test in GraphPad Prism. *, **, *** and **** indicate $P < 0.05$, $P < 0.01$, $P < 0.001$ and $P < 0.0001$, respectively, and ns indicates no significance.

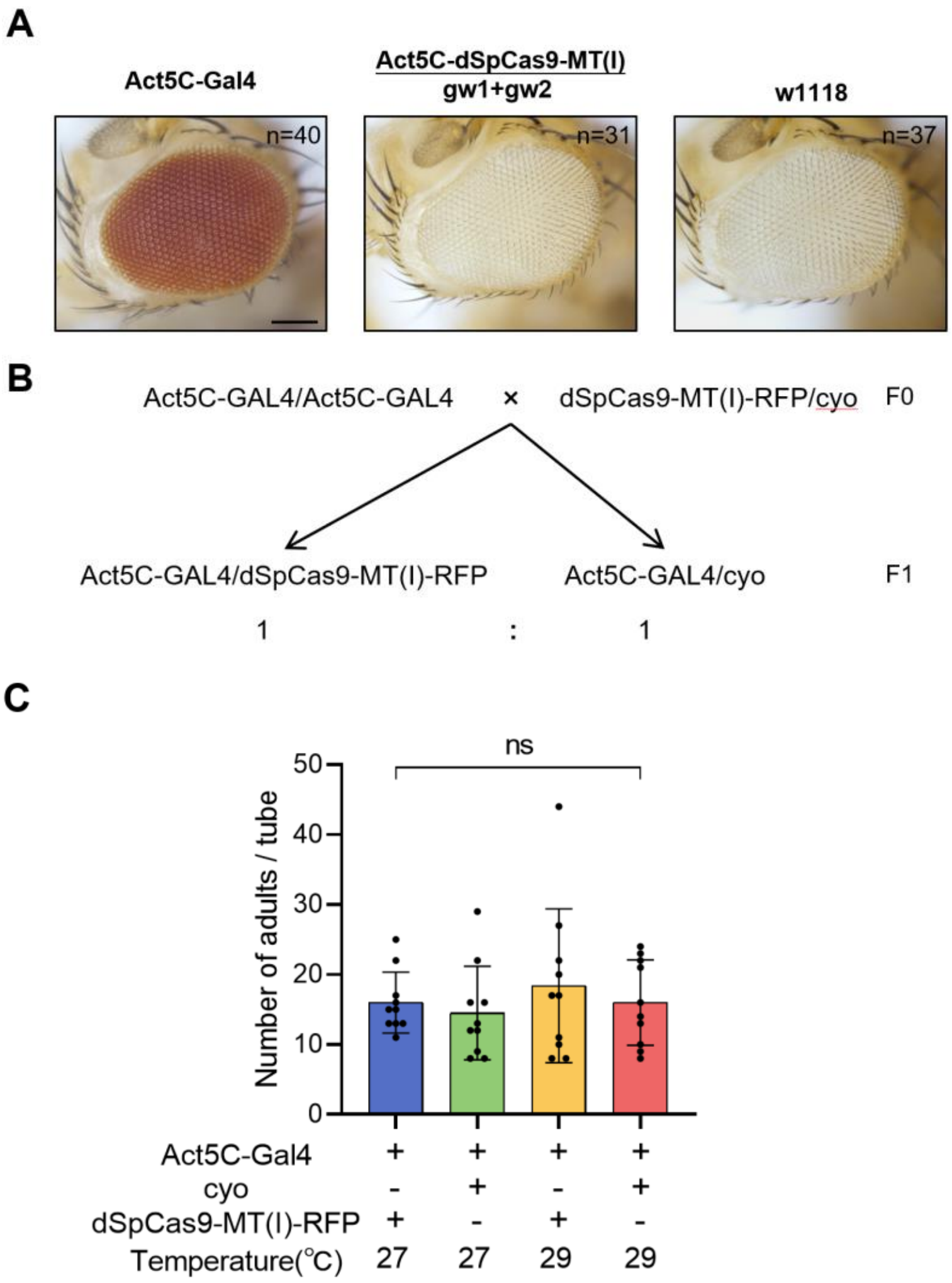

Figure S8. Analysis of dSpCas9-MT(I) fusion protein and eclosion viability test

A) Comparison of *w* gene phenotype defects caused by dSpCas9-MT(I) fusion protein with those in completely inactivated white gene fruit flies (w1118). Shown here are the eye structures of *Drosophila* captured using a macro zoom microscope. Images are representative of biological replicates, with the specific sample size (n) for each group indicated in the corresponding panels. Scale bar, 100 μm.

B) Schematic of the crosses designed for the lethality test experiment. In the absence of lethality, the expected ratio of F1 offspring containing RFP to those without RFP is 1:1.

C) Statistical comparison of the number of adult offspring per tube across different genotypes and culture temperatures. Each data point represents the total number of adults collected from a single independent cross (tube). Data are presented as mean ± SD (n =10 tubes per group). Statistical analysis was performed using one-way ANOVA with Tukey's honestly significant difference (HSD) test in GraphPad Prism. ns = not significant.

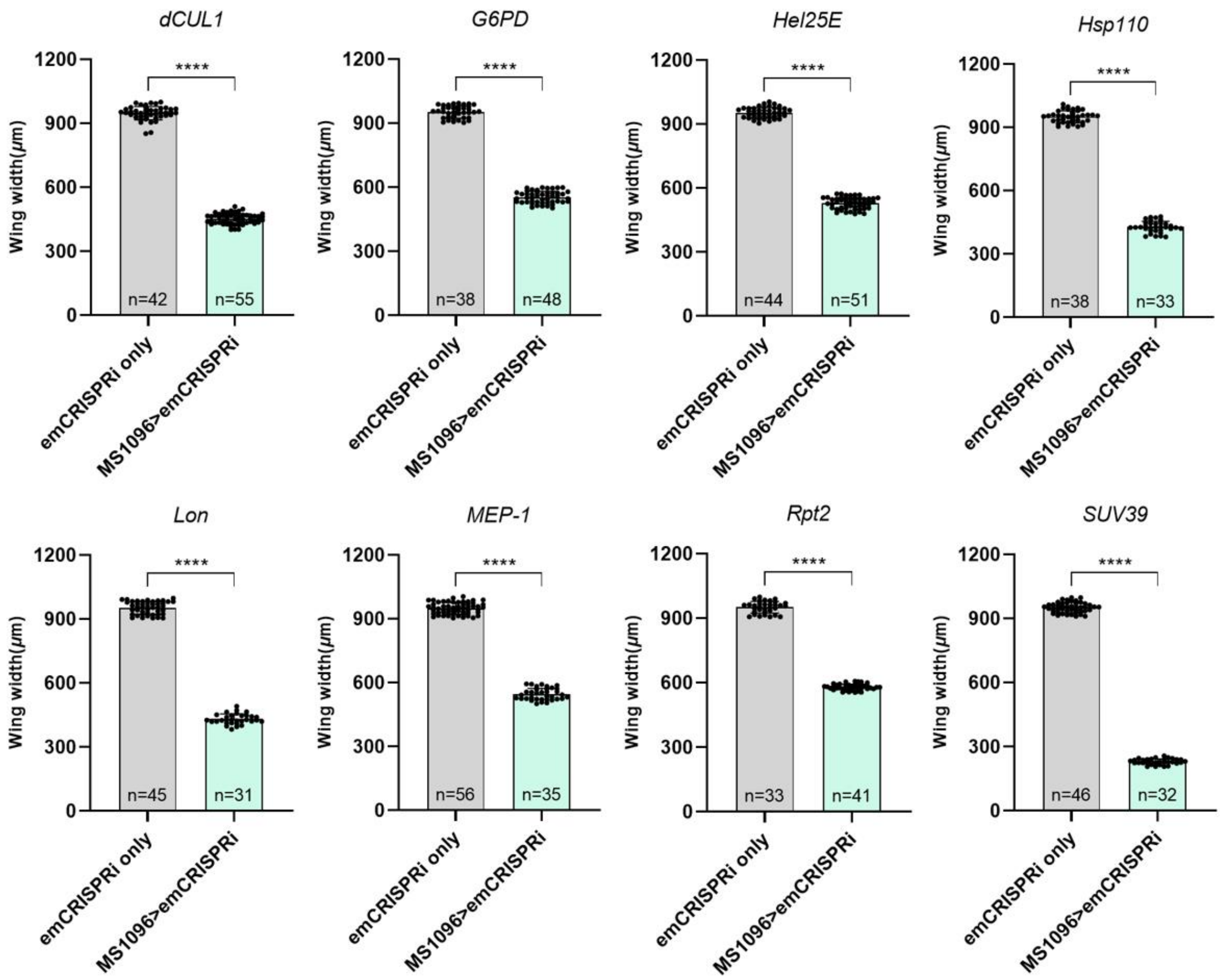

Figure S9. emCRISPRi induces significant phenotypic defects in *Drosophila* wings

Graph shows the comparison of wing width in emCRISPRi only flies and MS1096-GAL4 drived emCRISPRi flies. Data were analyzed using a two-tailed unpaired t-Test in GraphPad Prism. Data plotted as mean ± SD, with sample sizes (n) for each group indicated in the figure panels. ****, $p < 0.0001$.

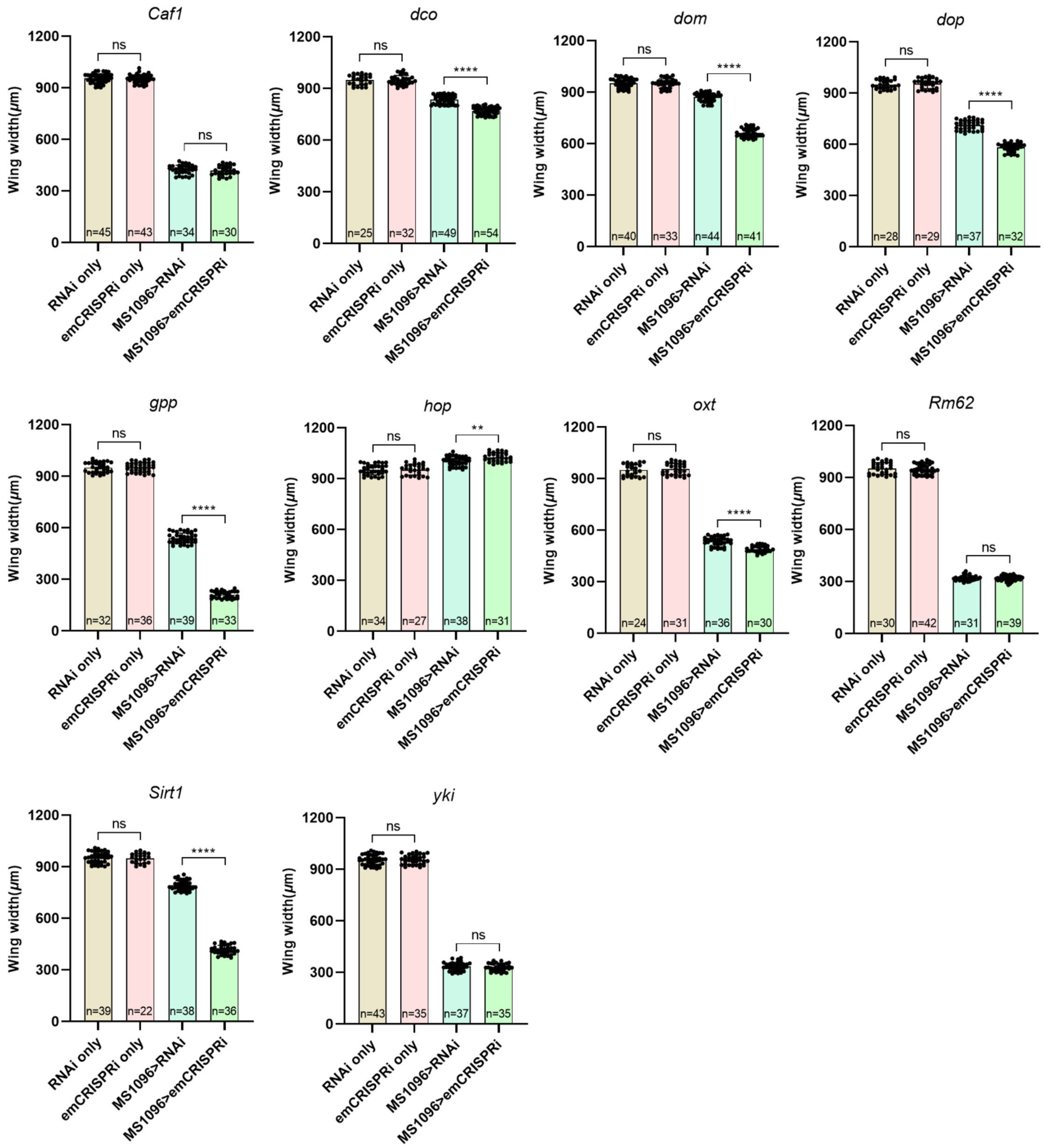

Figure S10. Both RNAi and emCRISPRi can induce significant phenotypic defects in *Drosophila* wings

Graph shows the comparison of wing width in RNAi only flies, emCRISPRi only flies, MS1096-GAL4 drived RNAi flies and emCRISPRi flies. Data were analyzed using a two-tailed unpaired t-Test in GraphPad Prism. Data plotted as mean ± SD, with sample sizes (n) for each group indicated in the figure panels. ****, $p < 0.0001$.

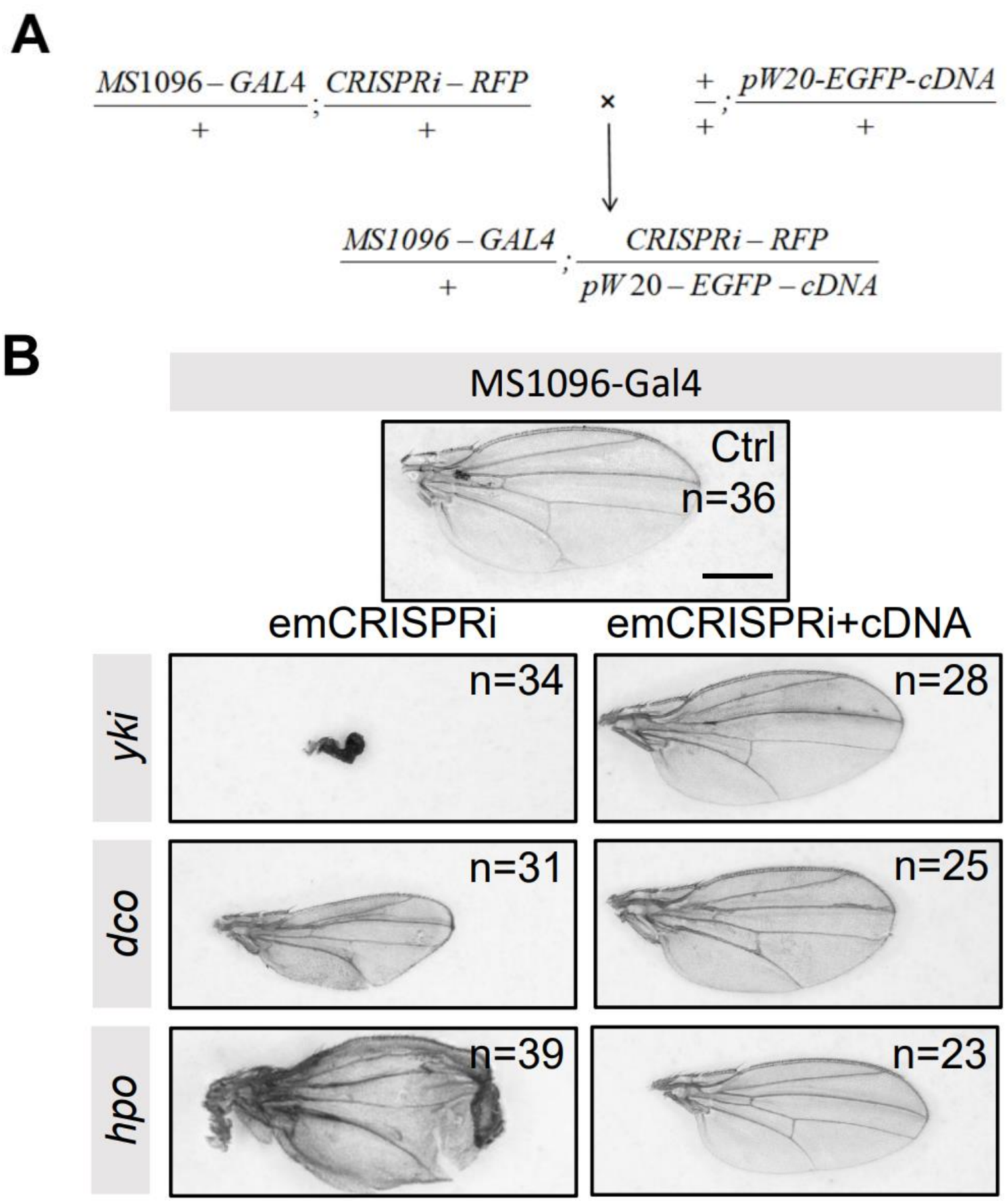

Figure S11. Schematic of the cDNA rescue experiment for emCRISPRi

A) A schematic of the crosses for rescuing phenotypic defects caused by emCRISPRi using cDNA, under the control of MS1096-Gal4.

B) Under the condition that emCRISPRi inhibits genes, cDNA was used to rescue the phenotypic defects caused by emCRISPRi. Shown here are the wings of *Drosophila* captured using a macro zoom microscope. Images are representative of biological replicates, with the specific sample size (n) for each group indicated in the corresponding panels. Scale bar, 500 μm. (Ctrl is *MS1096-Gal4/+*)

A

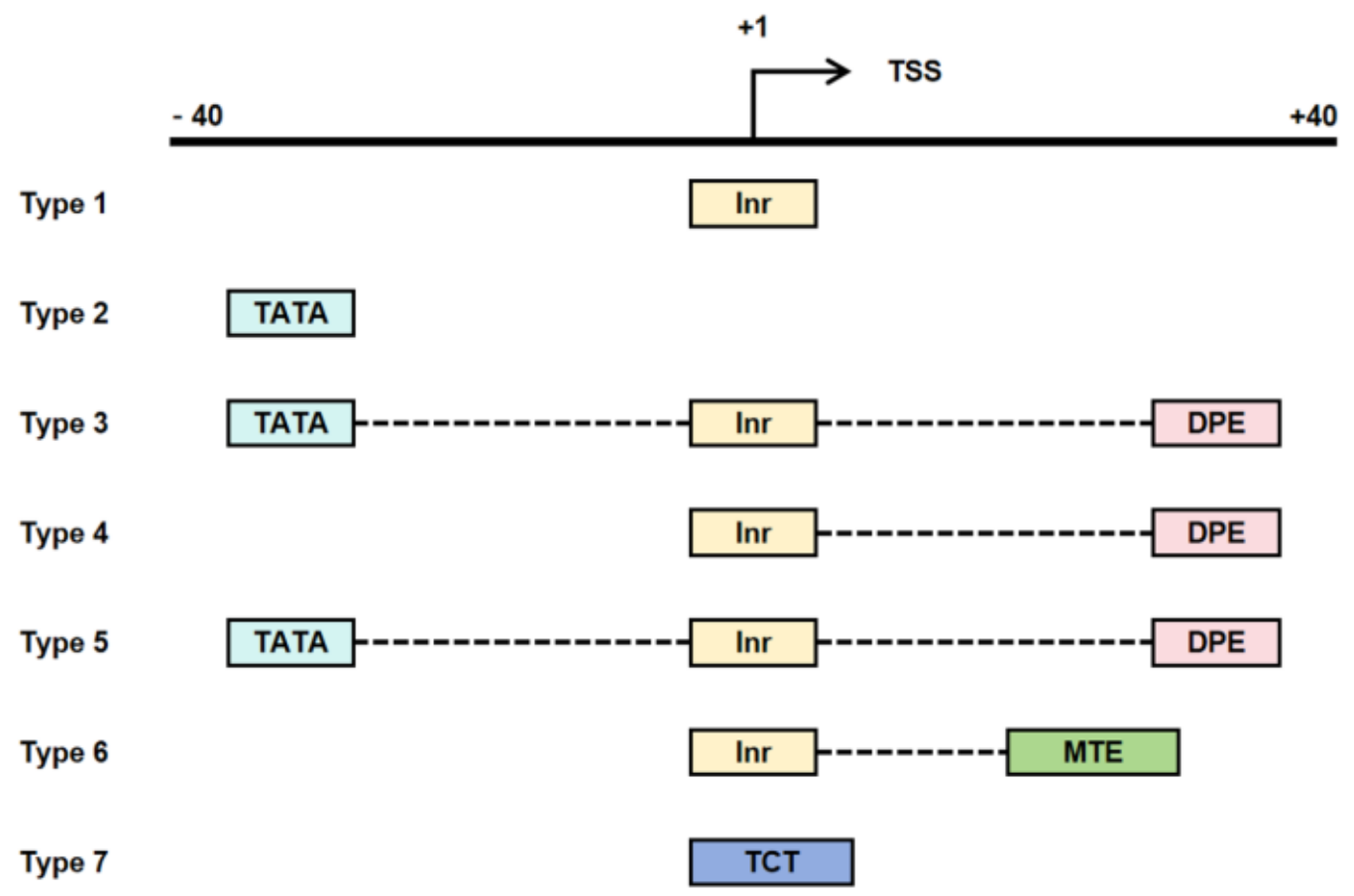

B

Type	Gene	TATA		TCT	Inr	MTE	DPE
		TATA-box	TATA-like				
1	*Rm62*				TCACTC		
1	*hpo*				CTAGTG		
2	*dom*		TGGCATTTC				
2	*G6PD*	TATAACTA					
3	*Caf1-55*		CTCTGTTAT		GCAGTC		
3	*dop*		GATAACTTG		CTAGTA		
3	*Lon*	AATAGAAG			TCAGCC		
4	*Sirt1*				CCAGCC		GCCATCTT
4	*Su(var)3-9*				CCAATC		TTGATGCC
5	*yki*		TTACATGAG		CAAGTG		GGGATAAA
5	*Hel25E*		CGATATCAC		TCACAC		CGCATCAC
6	*Oxt*				GTAGTA	CGAGCGGGGAGA	
6	*Rpt2*				TCATGT	CTAACGCGAATG	
7	*gpp*			TTGTTTCT			
7	*dco*			TTCGTTTA			

C

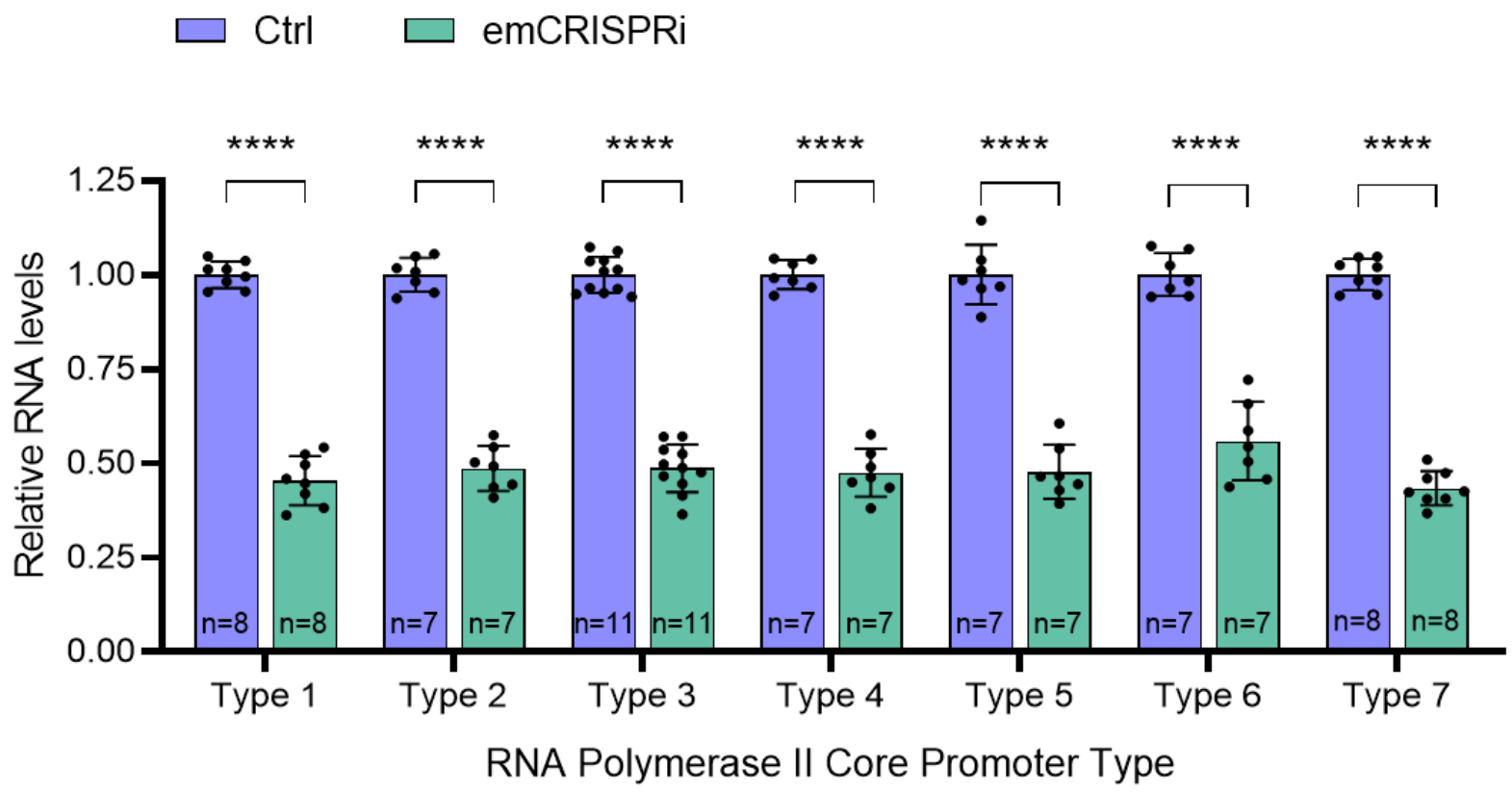

Figure S12. emCRISPRi can achieve transcriptional repression of various types of promoters

A) Some types of RNA Pol II promoters in *Drosophila*.

B) DNA motif sequences in RNA Pol II core promoters of *Drosophila* genes targeted in Fig. 2 and Fig. 3.

C) enCRISPRi exhibits strong inhibitory effects on multiple types of core promoters. The statistical data were compiled from the data in Figure 2 and Figure 3. Each data point in the bar graph represents an independent biological replicate, derived from the average of two technical replicates. Data are presented as mean ± SD, with sample sizes (n) for each group indicated in the figure panels. P values were determined using a two-tailed unpaired T-test in GraphPad Prism. ****, $P < 0.0001$.

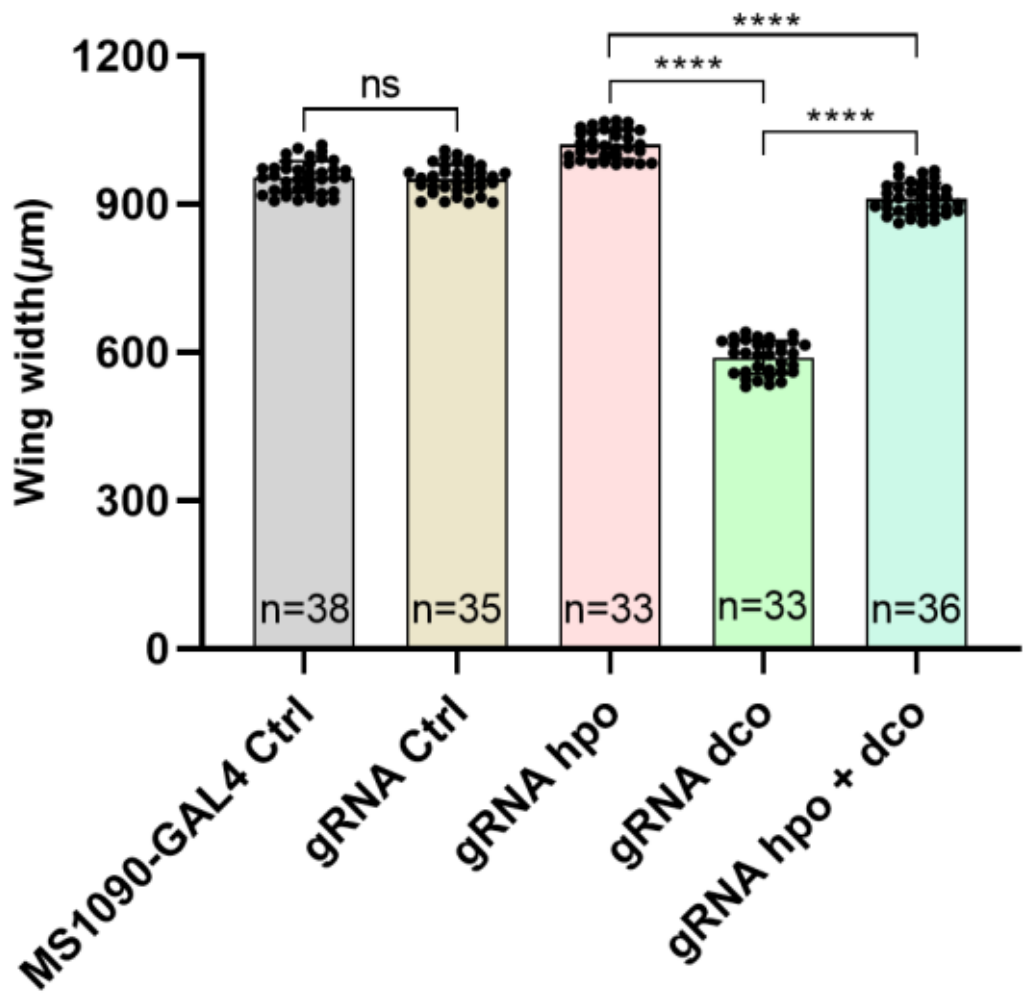

Figure S13. Application of emCRISPRi system in signal pathways

Graph shows the comparison of wing width in control flies and various gRNA flies. Statistical analysis was performed using one-way ANOVA with Tukey's honestly significant difference (HSD) test in GraphPad Prism. Data plotted as mean ± SD, with sample sizes (n) for each group indicated in the figure panels. ****, $p < 0.0001$, ns = not significant.

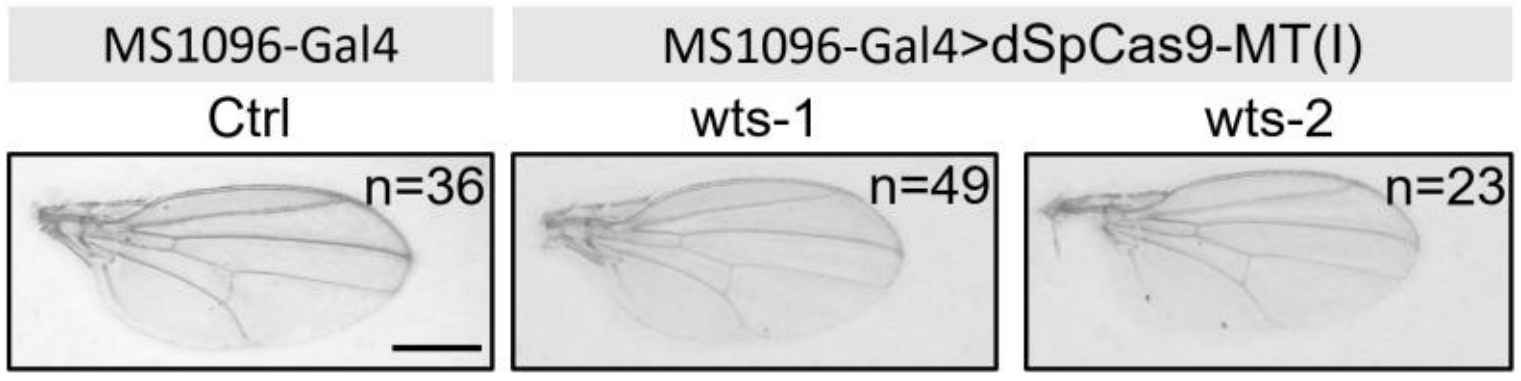

Fig. S14. The wts-1 and wts-2 gRNAs failed to produce obvious phenotypic defects. Neither wts-1 nor wts-2 gRNA induced a clear phenotype. Images are representative of biological replicates, with the specific sample size (n) for each group indicated in the corresponding panels. Scale bar, 500 μm. (Ctrl is *MS1096-Gal4/+*)
All flies were maintained at 27°C.

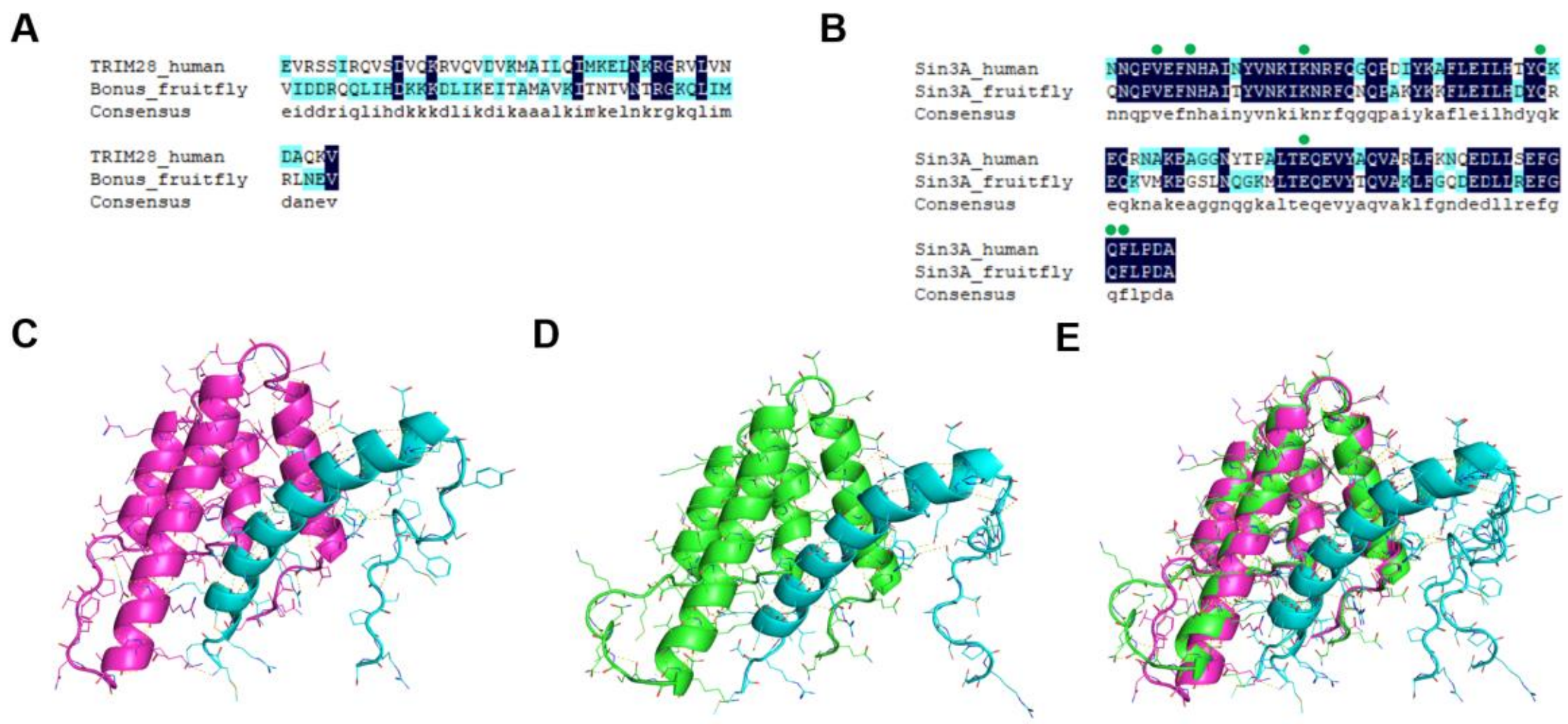

Figure S15. Sequence and structure alignment of TRIM28 and Sin3A homologues.

A) The KRAB-interacting region of TRIM28 was compared with its corresponding sequence in Bonus. The sequence alignment tool is DNAMAN.

B) The Mxi-interacting region of hSin3A was compared with its corresponding sequence in dSin3A. Green dots indicate the AlphaFold3-predicted interaction sites for Mxi on the Sin3A sequence. The sequence alignment tool is DNAMAN.

C) AlphaFold3 predicts the structure of the Mxi and hSin3A(partial) interaction. Purple: hSin3A, Marigold blue: Mxi.

D) AlphaFold3 predicts the structure of the Mxi and dSin3A(partial) interaction. Green: dSin3A, Marigold blue: Mxi.

E) Alignment of structures in figures c and d. Purple: hSin3A, Green: dSin3A, Marigold blue: Mxi. The structural alignment tool is PyMOL.
